# Covalent Adaptable Networks with Tailorable Material Properties Based on Divanillin Polyimines

**DOI:** 10.1021/acs.biomac.3c01224

**Published:** 2024-03-18

**Authors:** Noé Fanjul-Mosteirín, Karin Odelius

**Affiliations:** Wallenberg Wood Science Center, WWSC, Department of Fibre and Polymer Technology, KTH Royal Institute of Technology, SE-100 44 Stockholm, Sweden

## Abstract

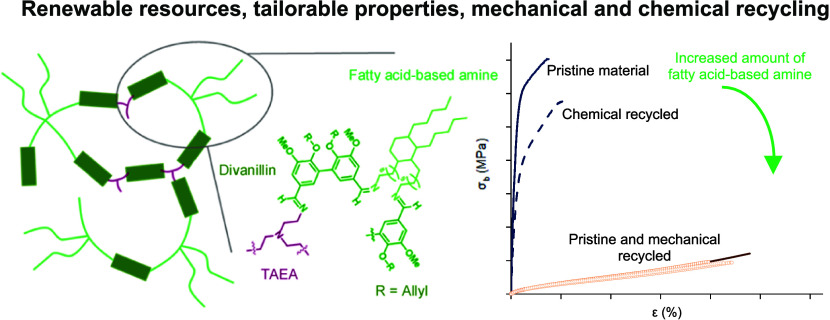

Covalent adaptable networks (CANs) are being developed as future replacements for thermosets as they can retain the high mechanical and chemical robustness inherent to thermosets but also integrate the possibility of reprocessing after material use. Here, covalent adaptable polyimine-based networks were designed with methoxy and allyloxy-substituted divanillin as a core component together with long flexible aliphatic fatty acid-based amines and a short rigid chain triamine, yielding CANs with a high renewable content. The designed series of CANs with reversible imine functionality allowed for fast stress relaxation and tailorability of the thermomechanical properties, as a result of the ratio between long flexible and short rigid amines, with tensile strength (σ_b_) ranging 1.07–18.7 MPa and glass transition temperatures ranging 16–61 °C. The CANs were subsequently successfully reprocessed up to three times without determinantal structure alterations and retained mechanical performance. The CANs were also successfully chemically recycled under acidic conditions, where the starting divanillin monomer was recovered and utilized for the synthesis of a recycled CAN with similar thermal and mechanical properties. This promising class of thermosets bearing sustainable dynamic functionalities opens a window of opportunity for the progressive replacement of fossil-based thermosets.

## Introduction

Thermosets are high-performance cross-linked materials that compared to thermoplastics display improved thermomechanical properties and chemical resistance. Although these advantages come with an inherent limitation, thermosets cannot be reprocessed after being cured, making mechanical recycling impossible, thereby reducing their applicability in a circular plastic economy.^1^ In order to fill the gap between the two material groups, covalent adaptable networks (CANs) arise as interesting candidates.^2−7^ CANs rely on the ability of certain chemical functionalities to undergo bond exchange upon external stimulus such as heat or light. Two different mechanisms have been proposed and proven, that is, dissociative CANs where the cross-linkers are divided into individual units before a subsequent rearrangement takes place and associative CANs in which the formation of a new bond takes place at the same time as another bond breaks. One of the benefits of the associative mechanisms is considered to be the small variation in cross-linking density regardless of the external stimuli, in comparison with the dissociative CANs.

A plethora of different chemistries for associative CANs has been introduced and includes transesterification reaction,^8^ transcarbamoylation reactions involving urea, urethane and hydroxy-urethane moieties,^9,10^ disulfide exchange,^11,12^ vinylogous urethane exchange,^13^ boronic ester metathesis,^14^ olefin metathesis,^15^ and imine metathesis.^16^ These chemistries all portray benefits and deficiencies, where the imine formation is known to be easily hydrolyzed under acidic conditions, enabling efficient chemical recycling by recovering original aldehydes. Also, due to the fast kinetics of the exchange reactions taking place in the network, self-healing behavior and fast stress relaxation has been observed in this class of CANs.^17^ In addition, the biobased pool of aldehydes is rather extensive, and includes vanillin,^18−20^ syringaldehyde,^21,22^ furfural,^23−25^ and functionalized lignin.^26−28^ Imine formation is a well-known reaction in which a primary amine reacts with an aldehyde, yielding a C=N moiety, with water as the sole byproduct.^29^ Taken together, imine-based CANs represent a promising platform as a substituent for nonrenewable materials since dynamic covalent chemistries allow closed-loop recyclability.^30^ As thermosets and their future replacements in the form of CANs are commonly utilized for long-term applications, transforming the products recourse from a fossil-based to a biobased resource also enables their use as a carbon sink during their complete lifetime.

Vanillin is a very interesting renewable building block having both a phenolic unit along with an aldehyde and it has been exploited for the synthesis of a large plethora of different types of polymers.^31,32^ The phenol moiety can easily be functionalized with many different electrophiles, and the aldehyde functionality opens the possibility for the preparation of polyimine-based CANs.^17^ For example, vanillin was first methacrylated on the phenol prior to being reacted with two different amines, 2,2′-(ethylenedioxy)bis(ethylamine) and trimethylolpropane tris[poly(propylene glycol), amine terminated] ether (Jeffamine), where the latter provided increased flexibility. The imine-methacrylated monomers were rapidly photo-cross-linked, and the obtained networks showed the expected behavior with fast stress-relaxation and chemical recyclability as a consequence of their imine exchange ability.^33^ Phosphorus-containing polymers are known to possess flame-retardant properties,^34^ and in combination with vanillin, a sustainable platform for the preparation of several phosphorus-based polymers featuring flame-retardant properties has been proven.^35−37^ In addition, vanillin has been exploited for the synthesis of hybrid epoxy-imine thermosets via functionalization of the phenol moiety with epichlorohydrin, followed by curing with different amines^38−40^ and has been used as a platform in the fabrication of polyurethane resins by the coupling between the phenol moiety of vanillin with various isocyanates.^41−43^

Divanillin, Di-Van, contrary to vanillin, inherently have two aldehyde groups in their structures and are obtained via oxidative dimerization of vanillin. The bulky structure Di-Van contributes with high rigidity as a consequence of a reduced rotational motion of the structure directly linked through 5 and 5′ positions and the lack of any spacers. Therefore, an improvement of the thermodynamic performance has been reported compared to when vanillin was used as core.^44,45^ Di-Van has previously been utilized as a replacement for bisphenol A in an epoxy resin, by first reacting it with epichlorohydrin rendering epoxidized divanillin (EDV) and subsequently cured by diamines, generating a mixture of imine, amide, and ester cross-links with resins portraying similar thermomechanical properties to the commercial bisphenol A-based epoxy resin.^46^ Here, we aim to utilize the structure of Di-Van to design a simple synthetic approach achieving imine-chemistry-based CANs with a high biobased content, simple reprocessability, and chemical recyclability with a broad range of thermal and mechanical properties. To achieve this, we combine di- and triamines of different chemical structures with two different functionalized Di-Van monomers, which ultimately should provide different flexibility to the CAN as a consequence of the substituents length. The thermomechanical properties were successfully tailored by using different ratios of di- and triamines, and the presence of reversible imine functionalities in the network allowed both reprocessability of the prepared materials, proved via stress-relaxation experiments, along with the possibility of chemical recycling under aqueous acidic conditions and resynthesis of a CAN with similar thermal and mechanical properties.

## Experimental Section

### Materials

All chemicals were used as received without further purification unless otherwise indicated. This includes vanillin (Sigma-Aldrich, 99%), Mohr salt (Sigma-Aldrich, 99%), potassium persulfate (Sigma-Aldrich, 99%), potassium carbonate (Sigma-Aldrich, 99%), iodomethane (Sigma-Aldrich, 99%), allyl bromide (Sigma-Aldrich, 99%), and tris(2-aminoethyl)amine (Sigma-Aldrich, 96%). Priamine 1071 was kindly supplied by Croda, DMF (VWR, 99.8%).

### Characterization

NMR spectra were recorded on a Bruker Avance 400 MHz spectrometer. Chemical shifts are reported in parts per million (ppm) and referenced to the residual solvent signal (CDCl_3_: ^1^H, δ = 7.26 ppm, ^13^C, δ = 77.2 ppm, DMSO: ^1^H, δ = 2.50 ppm, ^13^C, δ = 39.5 ppm, TMS: ^1^H, δ = 0.00 ppm). Multiplicities are reported as s = singlet, brs = broad singlet, d = doublet, t = triplet, and m = multiplet. Multiplicity is followed by integration and coupling constant (*J*) in Hz.

FTIR spectra were recorded on a PerkinElmer Spectrum 2000 instrument equipped with a single reflection attenuated total reflectance (ATR) accessory. 8 to 16 scans were recorded with a 4 cm^–1^ resolution, between 4000 and 600 cm^–1^.

Dynamic mechanical analysis (DMA) of the covalent adaptable networks (CANs) were measured with a Q800 dynamic mechanical analyzer (TA Instruments) in tension mode. Rectangular-shaped specimens were taken from hot-pressed samples. Determination of *T*_g_ was carried out by triplicate with a 3 °C min^–1^ heating rate and 0.1% strain at 1 Hz. Stress-relaxation experiments were carried out with a 2% strain for 20 min. Frequency sweep experiments were carried out at room temperature with a 0.1% strain and a logarithmic frequency sweep from 0.1 to 100 Hz. Cross-linking density (υ_e_) was calculated using eq 1, where *E*′ is the storage modulus at the rubbery plateau at the respective temperature *T* and *R* is the universal gas constant (8.314 J K^–1^ mol^–1^). DMA data were analyzed by TA Universal Analysis Software (v. 4.5).
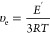
1

Compression molding of the CANs was carried out on a TP 400 hot press (Fontijne Presses BV). Each CAN was first ground with a coffee grinder and then dispersed in a steel mold with the appropriate shape (dumbbell or strip). The mold was sandwiched between two stainless steel disks (Ø = 23 cm, *T* = 0.5 cm) covered with thin PTFE sheets (*T* = 0.1 mm). The samples were pressed at 140 °C for 20 min with several (at least three) venting cycles.

The gel content (*GC*) of the synthesized CANs was calculated through eq 2
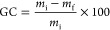
2

A piece of each CAN with initial mass *m*_i_ was immersed in the corresponding solvent for 24 h. After, the solvent was decanted off, and the CAN was dried under reduced pressure until constant mass *m*_f_ was reached. The measurement was repeated three times for each network.

Tensile testing was performed on an Instron 5944 tensile tester. The CANs were tested using dumbbell-shaped specimens (38 mm (*L*) × 5 mm (*W*) × 0.8 mm (*T*), effective gauge length 22 mm) prepared by hot-pressing the ground CANs in a custom-made steel mold with the same dimensions. The cross-head speed was set to 0.05 mm·min^–1^.

The thermal stability of the synthesized CANs was evaluated by a Mettler Toledo TGA/DSC 851e module instrument. An inert flow (nitrogen) of 50 mL/min and a heating rate of 5 °C per minute was utilized. The temperature scan was performed from 25 to 650 °C. The onset temperatures (*T*_onset_) at 5 wt % mass loss were determined.

### Synthesis of Divanillin (Di-Van)

Vanillin (15 g, 98.60 mmol, 1 equiv) was dissolved in water (600 mL) until total dissolution at 90 °C. (NH_4_)_2_Fe(SO_4_)_2_·6 H_2_O (1.7 g, 4.34 mmol, 0.044 equiv) and K_2_S_2_O_8_ (16 g, 59.15 mmol, 0.600 equiv) were added, the heating was kept for additional 30 min, turned off, and stirred for additional 10 min. The precipitated product was filtered and dissolved in 5 M NaOH (100 mL) and then reprecipitated by addition of 5 M HCl (100 mL). This solid was filtered on a Büchner funnel and washed with large amounts of boiling distilled water, followed by boiling MeOH (14.5 g obtained, 47.97 mmol, 97% yield). Characterization data was in agreement with those reported in the literature.^47^^**1**^**H NMR** (400 MHz, DMSO-d_6_) δ 9.81 (bs, 4H, C*H*O, O*H*), 7.57 (d, 4H, ^3^*J*_H–H_ = 2.5 Hz, H_Ar_), 3.93 (s, 3H, OCH_3_). ^**13**^**C APT NMR** (100 MHz, DMSO-d_6_) δ 191.6 (CHO), 150.4 (C–OH), 148.2 (*C*-OCH_3_), 128.2 (CH), 127.8 (*C*-CHO), 124.6 (C–C), 109.1 (CH), 56.0 (OCH_3_).

### Synthesis of Di-Van-OMe

Divanillin (14.06 g, 46.51 mmol, 1 equiv) was dissolved in DMF (232 mL). Potassium carbonate (27.19 g, 196.75 mmol, 4.23 equiv) was added before a slow addition of iodomethane (8.8 mL, 141.40 mmol, 3.04 equiv). The mixture was stirred overnight at 80 °C; then, the mixture was filtered and the liquors were poured into cold water; a solid was precipitated and filtered over a Büchner funnel and further dried under high vacuum (11.15 g obtained, 33.75 mmol, 73% yield). Characterization data was in agreement with those reported in the literature.^47^^**1**^**H NMR** (400 MHz, DMSO-d_6_) δ 9.93 (s, 2H, CHO), 7.58 (d, 2H, ^3^*J*_H–H_ = 1.9 Hz, H_Ar_), 7.45 (d, 2H, ^3^*J*_H–H_ = 1.9 Hz, H_Ar_), 3.93 (s, 3H, OCH_3_), 3.67 (s, 3H, OCH_3_). ^**13**^**C APT NMR** (100 MHz, DMSO-d_6_) δ 192.3 (CHO), 153.3 (*C*-OCH_3_), 152.0 (*C*-OCH_3_), 132.3 (*C*-CHO), 132.0 (C–C), 126.6 (CH), 111.8 (CH), 60.9 (OCH_3_), 56.5 (OCH_3_).

### Synthesis of Di-Van-OAllyl

Divanillin (7.50 g, 24.81 mmol, 1 equiv) was dissolved in DMF (124 mL). Potassium carbonate (20.60 g, 148.87 mmol, 6 equiv) was added before a slow addition of allyl bromide (9.4 mL, 109.17 mmol, 4.4 equiv). The mixture was stirred overnight at 80 °C; then, the mixture was filtered, the liquors were poured into cold water, and a solid was precipitated and filtered over a Büchner funnel and further dried under high vacuum (7.06 g obtained, 18.51 mmol, 74% yield). Characterization data agreed with those reported in the literature.^48^^**1**^**H NMR** (400 MHz, CDCl_3_) δ 9.90 (s, 2H, CHO), 7.49 (d, 4H, ^3^*J*_H–H_ = 1.9 Hz, H_Ar_), 7.42 (d, 4H, ^3^*J*_H–H_ = 1.9 Hz, H_Ar_), 5.80–5.70 (m, 2H, C*H*=CH_2_), 5.10–5.02 (m, 4H, CH=C*H*_2_), 4.48 (d, 4H, ^3^*J*_H–H_ = 5.7 Hz, OC*H*_2_-CH=CH_2_), 3.97 (s, 6H, OCH_3_). ^**13**^**C APT NMR** (100 MHz, CDCl_3_) δ 191.3 (CHO), 153.7 (*C*-OCH_2_–CH=CH_2_), 151.5 (*C*-OCH_3_), 133.8 (*C*H=CH_2_), 132.3 (*C*-CHO), 132.2 (C–C), 128.2 (CH), 117.9 (CH=*C*H_2_), 110.3 (CH), 74.4 (O*C*H_2_–CH=CH_2_), 56.3 (OCH_3_).

### Synthesis of CANs MeO-Pri_*x*_-TAEA_*y*_ and AllylO-Pri_*x*_-TAEA_*y*_

To a solution of Priamine 1071 (0.33–1 equiv) and tris(2-aminoethyl)amine (0.66–0 equiv) in CH_2_Cl_2_ (2 mL), a solution of monomer **Di-Van-OMe** (2 g, 6.05 mmol, 1 equiv) or **Di-Van-OAllyl** (2 g, 5.24 mmol, 1 equiv) in CH_2_Cl_2_ (12 mL) was added. The mixture was stirred in a vortex for 1 min and then poured in a Teflon mold (10 cm diameter). The solvent was left to evaporate at room temperature overnight, and a film was obtained. The obtained film **MeO-Pri**_**x**_**-TAEA**_**y**_ or **AllylO-Pri**_**x**_**-TAEA**_**y**_ was cured in an oven at 140 °C for 8 h.

### Synthesis of Linear Diimine Derived from Di-Van-OMe and Octadecylamine

In a 4 mL crimped vial, **Di-Van-OAllyl** (200 mg, 0.52 mmol, 1 equiv) and octadecylamine (290 mg, 1.07 mmol, 2.05 equiv) were heated for 8 h at 140 °C. A brown solid was obtained. ^1^H NMR (400 MHz, CDCl_3_) δ 8.17 (s, 1H, CH=N), 7.46 (d, 4H, ^3^*J*_H–H_ = 2.0 Hz, H_Ar_), 7.11 (d, 4H, ^3^*J*_H–H_ = 1.9 Hz, H_Ar_), 5.83–5.73 (m, 2H, C*H*=CH_2_), 5.11–4.98 (m, 4H, CH=C*H*_2_), 4.37 (d, 4H, ^3^*J*_H–H_ = 5.8 Hz, OCH_2_–CH=CH_2_), 3.95 (s, 6H, OCH_3_), 3.58 (t, 4H, ^3^*J*_H–H_ = 6.8 Hz, CH=N–C*H*_2_), 1.71–1.65 (m, 4H, CH=N–CH_2_–C*H*_2_-), 1.33–1.25 (m, 60H, N–CH_2_–CH_2_–(C*H*_2_)_15_-CH_3_), 0.88 (t, 6H, ^3^*J*_H–H_ = 6.8 Hz, N–CH_2_–CH_2_–(CH_2_)_15_–C*H*_3_).

## Results and Discussion

In order to obtain a rigid biobased chemical platform to be used in the synthesis of CANs, vanillin oxidative dimerization followed by functionalization was performed. The divanillin precursors were synthesized to have retained aldehyde functionality, enabling reactions with amines, yielding the corresponding imines with a well-known reversible behavior. The CANs were designed to achieve a high biobased content and to tailor the thermal and mechanical properties by combining rigid moieties with long and aliphatic flexible fatty acid-based amines. The fatty acid-based amine, Priamine 1071 (**Pri**), is a cross-linking agent extracted from vegetable oils such as soybean and sunflower oil which as a consequence of its high content of aliphatic flexible chains confers both elasticity and hydrophobicity.^49^

**Scheme 1:**
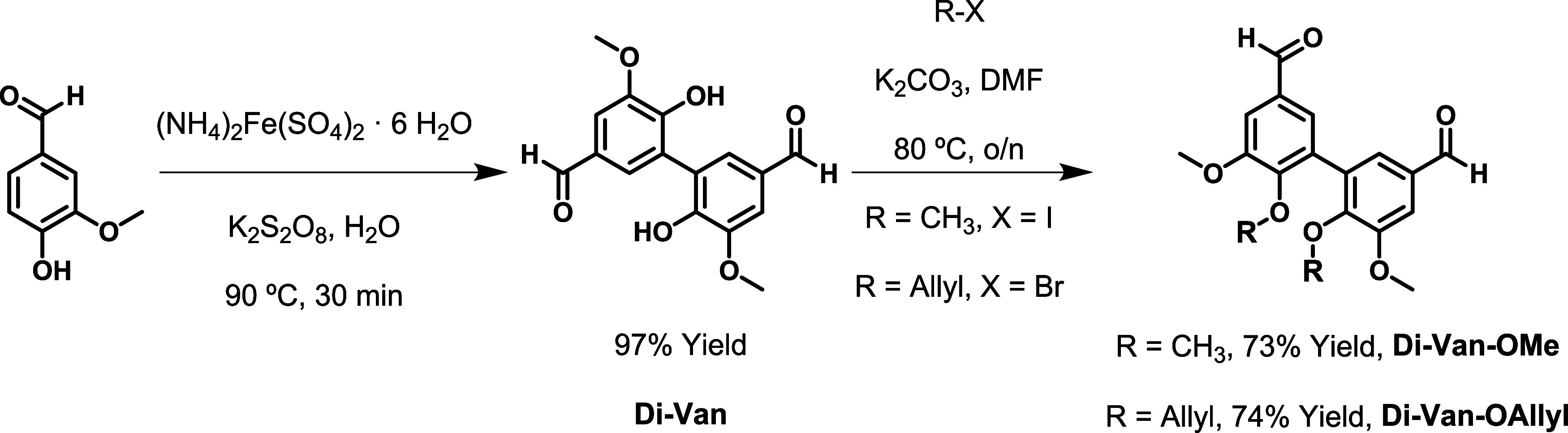
Vanillin Dimerization Followed by Phenol Functionalization

Divanillin (**Di-Van)** was obtained at excellent yields via oxidative dimerization as previously described in the literature.^50^ As **Di-Van** has a low solubility and two acidic phenol moieties that ultimately can react in an acid–base process with amines leading to secondary reactions and therefore preventing film-casting by simple reactant mixing at room temperature or via the use of using low boiling point solvents, functionalization of the **Di-Van** phenol moieties rendered two different monomers with increased solubility, **Di-Van-OMe** and **Di-Van-OAllyl**. The choice of functionalization was made to achieve a comparison between the **Di-Van** monomers with a more rigid and short methoxy or longer and more flexible allyloxy functionality. Both monomers were isolated at high yields, 73 and 74%, respectively (Scheme 1).

**Scheme 2:**
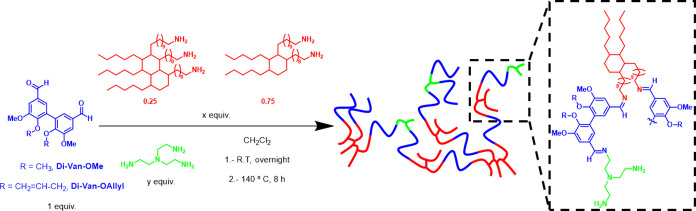
Synthesis of Divanillin-Based CANs **MeO-Pri**_**x**_**-TAEA**_**y**_ and **AllylO-Pri**_**x**_**-TAEA**_**y**_

By combining **Di-Van-OMe** or **Di-Van-OAllyl** with varying ratios of the di- and trifunctional fatty acid-based amine, **Pri** and tris(2-aminoethyl) amine (**TAEA**), six different CANs were designed, by film-casting at room temperature, followed by a thermal curing process at 140 °C for 8 h (Scheme 2). The CANs were named according to **MeO-Pri**_**x**_**-TAEA**_**y**_ and **AllylO-Pri**_**x**_**-TAEA**_**y**_, where x and y refer to the molar ratio with respect to the aldehyde monomer. As the ratios of fatty acid amine and **TAEA** were varied, CANs with different **Di-Van** functionality and cross-linking densities and ultimately different thermomechanical properties were created (Table 1).

**Table 1:** Thermal and Viscoelastic Properties of CANs **MeO-Pri**_**x**_**-TAEA**_**y**_ and **AllylO-Pri**_**x**_**-TAEA**_**y**_

entry	CAN	*T*_g_ (°C)^a^	*T*_d,5%_ (°C)	*E*_a_ (kJ/mol)^b^	*T*_v_ (°C)^b^	*E*′ (MPa)^c^	υ_e_ (mol m^–3^)^d^
1	MeO-Pri_1_-TAEA_0_	28 ± 2	369	51.3	–27	2.07 ± 0.70	235 ± 80
2	MeO-Pri_0.66_-TAEA_0.33_	34 ± 1	337	95.9	17	2.95 ± 0.18	325 ± 20
3	MeO-Pri_0.33_-TAEA_0.66_	61 ± 2	280	49.1	11	n.d	n.d
4	AllylO-Pri_1_-TAEA_0_	16 ± 2	338	46.9	–57	1.78 ± 0.11	202 ± 13
5	AllylO-Pri_0.66_-TAEA_0.33_	18 ± 4	292	72.6	–27	1.48 ± 0.36	168 ± 41
6	AllylO-Pri_0.33_-TAEA_0.66_	61 ± 2	269	39.6	–22	n.d	n.d

a Obtained by triplicate DMA measurements.

b Obtained from strain–stress-relaxation curves fitted to the Arrhenius law and adjusted to a Maxwell model.

c Storage modulus in the rubbery plateau.

d Calculated used equation .

To ensure that the longer and more flexible allyloxy functionality on **Di-Van** during CAN formation is unreactive against amines, a model reaction was performed. We here mimicked the curing conditions (140 °C for 8 h) and chose octadecylamine as the amine, as it bears a resemblance to the long flexible aliphatic chain of fatty acid amine. The ^1^H NMR spectrum of the reaction crude revealed the sole formation of the desired imine derivative, and no side reactions took place on the allyloxy functionality (Figure S7).

The formation of the desired imine CANs was confirmed by Fourier transformed infrared (FTIR) spectroscopy, where the aldehyde stretching band at 1691 and 1686 cm^–1^ for the monomers **Di-Van-OMe** or **Di-Van-OAllyl** respectively disappeared, whereas the characteristic imine stretching band at lower frequencies (1645–1643 cm^–1^) was observed (Figures S8–S15). All CANs showed higher thermal stability when compared to their starting monomers, for which the monomer **Di-Van-OMe** and **Di-Van-OAllyl** exhibit a *T*_d,5%_ value of 261 and 236 °C (Figure S16), whereas the CANs display a range of *T*_d,5%_ 269–369 °C as determined by thermogravimetric analysis (TGA). In general, the CANs prepared from the monomer **Di-Van-OMe** had a higher thermal stability compared to the ones prepared from monomer **Di-Van-OAllyl** as a consequence of the lower reactivity toward the expected thermal degradation processes taking place at high temperatures of the methoxy group compared to the allyloxy one. Higher ratios of fatty acid-based amine also provided higher thermal stability, when compared to **TAEA** (Table 1). The latter phenomenon can be attributed to the higher C/N content present in the fatty acid-based amines and therefore in the lower tendency for thermal degradation to occur on the amine moieties.^51^ The presence of the functionalized **Di-Van** moiety commonly provided higher thermal stability when compared with other imine-based CANs systems where the same fatty acid-based amine was used as the sole amine source. For example, a series of different imine-based CANs designed from oxidized organosolv lignin reacted with different ratios of the fatty acid-based amines portrayed a *T*_d,5%_ range of 298–323 °C, where higher ratios of fatty acid-based amine led to higher thermal stability.^27^

**Figure 1:**
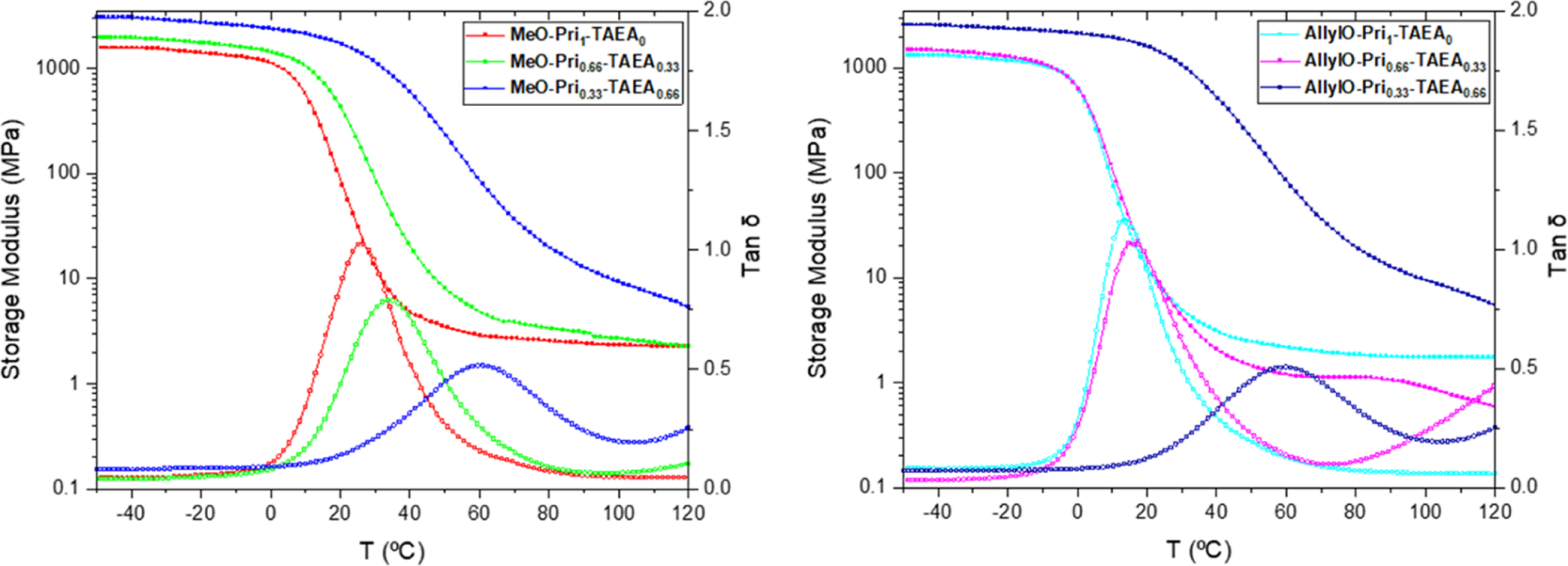
DMA curves of CANs **MeO-Pri**_**x**_**-TAEA**_**y**_ (*left*), **MeO-Pri**_**1**_**-TAEA**_**0**_ (*red*), **MeO-Pri**_**0.66**_**-TAEA**_**0.33**_ (*green*), **MeO-Pri**_**0.33**_**-TAEA**_**0.66**_ (*blue*), and **AllylO-Pri**_**x**_**-TAEA**_**y**_ (*right*), **AllylO-Pri**_**1**_**-TAEA**_**0**_ (*cyan*), **AllylO-Pri**_**0.66**_**-TAEA**_**0.33**_ (*purple*), and **AllylO-Pri**_**0.33**_**-TAEA**_**0.66**_ (*royal blue*).

The thermal transitions, viscoelastic behavior, and stress-relaxation behavior of the obtained CANs were determined by dynamic mechanical analysis (DMA). First, the evolution of storage (*E*′) and loss (*E*″) moduli was monitored between −50 and 120 °C and the *T*_g_ values were obtained from the maxima value of the tan δ curves (Figure 1). The CANs with the highest content (0.66 equiv) of the small and rigid amine **TAEA**, *i.e*., **MeO-Pri**_**0.33**_**-TAEA**_**0.66**_ and **AllylO-Pri**_**0.33**_**-TAEA**_**0.66**_, exhibited the highest *T*_g_ values, 61 °C for both **Di-Van** functionalities (Table 1, entries 3 and 6). Lower ratios of **TAEA** led to CANs with a lower *T*_g_ as a consequence of the increase of chain flexibility due to the higher amount of fatty acid-based amine (Table 1, entries 2 and 4). The influence of the substituents on the **Di-Van** phenol moiety were most evident in the CANs where none or low contents of **TAEA** was used (Table 1, entries 1, 2, 4, and 5), where the methoxy moiety confers higher rigidity to the cross-linked network and therefore the observed *T*_g_ values were higher when compared to the allyloxy counterparts. Unexpectedly, very similar *T*_g_ values were registered in the cases of CANs **AllylO-Pri**_**1**_**-TAEA**_**0**_ and **AllylO-Pri**_**0.66**_**-TAEA**_**0.33**_ with only 2 degrees of difference. In addition, it was observed that the cross-link density (υ_e_), calculated from the rubbery plateau, for the CANs bearing the **Di-Van-OMe** moiety revealed a lower υ_e_ = 235 ± 80 mol m^–3^ for **MeO-Pri**_**1**_**-TAEA**_**0**_ when compared to the more cross-linked **MeO-Pri**_**0.66**_**-TAEA**_**0.33**_ υ_e_ = 325 ± 20 mol m^–3^ (Table 1, entries 1 and 2). This is expected as the partial replacement of the long and highly flexible **Pri** with the short and rigid **TAEA** should result in a more cross-linked CAN with an expected higher *E*_a_. On the contrary, in the scenario of CANs based on the allyloxy moiety, the υ_e_ values do not follow the expected trend when higher amounts of **TAEA** was employed, and values of 168 ± 41 mol m^–3^ for the highly cross-linked **AllylO-Pri**_**0.66**_**-TAEA**_**0.33**_ and 202 ± 13 mol m^–3^ for **AllylO-Pri**_**1**_**-TAEA**_**0**_ (Table 1, entries 4 and 5) were obtained. Both CANs bearing the highest content of **TAEA** and therefore being the most rigid and the most cross-linked CANs, **MeO-Pri**_**0.33**_**-TAEA**_**0.66**_ and **AllylO-Pri**_**0.33**_**TAEA**_**0.66**_, did not show a rubbery plateau after the *T*_g_. Instead, the storage moduli keep decreasing, and at the same time an increase in the tan δ value reveals that the CANs are in the terminal zone where viscous dominance is present instead of elastic one (Table 1, entries 3 and 6). This is similar to what has previously been found for other polyimine-based CANs of various structures.^52−54^ As a consequence of the functionalized rigid **Di-Van** core, the observed *T*_g_ resulted to be higher when compared to other fully biobased systems where 2,5-furandicarboxyaldehyde was coupled with an slight excess (1.2 equiv) of the same fatty acid-based amine with CANs having a *T*_g_ of −10 °C by DSC analysis.^23^

**Figure 2:**
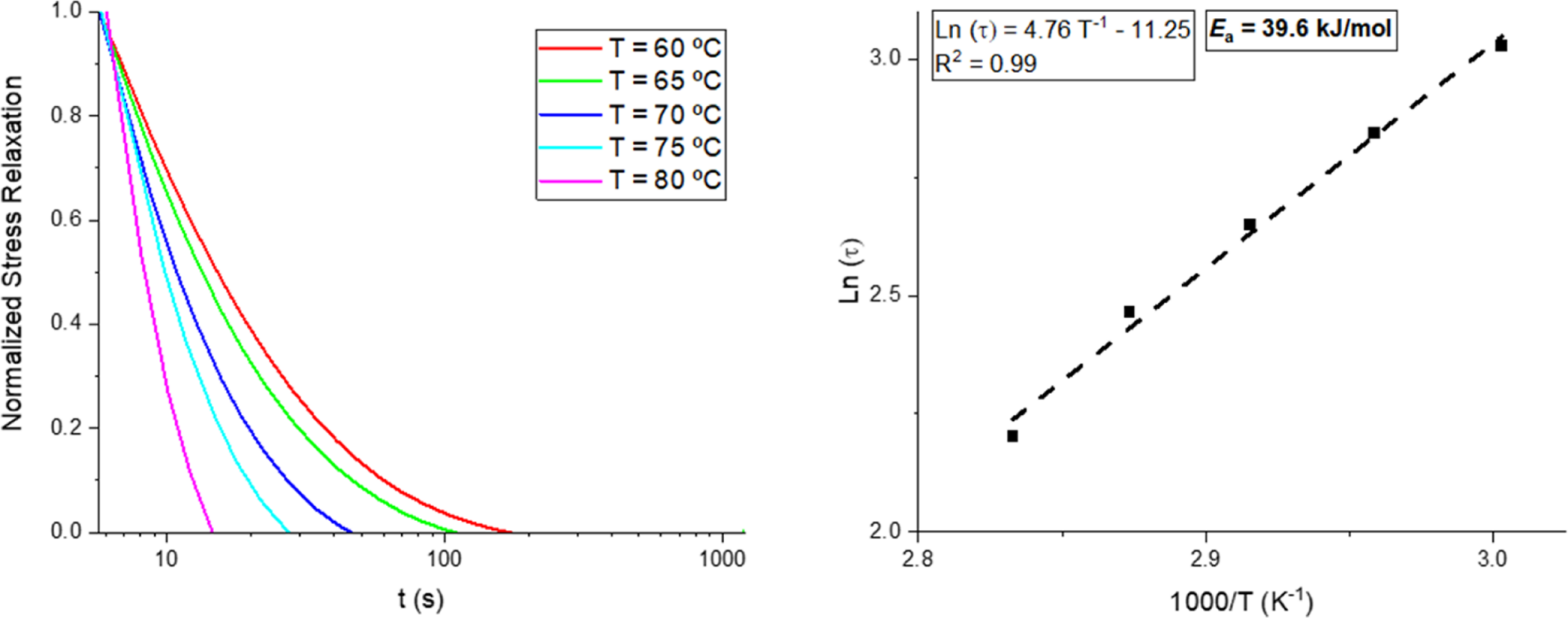
Stress-relaxation curve of **AllylO-Pri**_**0.33**_**-TAEA**_**0.66**_ (*left*) and Arrhenius plot obtained from the relaxation times τ* used to calculate the *E*_a_ of **AllylO-Pri**_**0.33**_**-TAEA**_**0.66**_ (*right*).

In order to prove the dynamic character of the prepared CANs, stress-relaxation experiments were performed. A constant strain of 2% was applied, and the relaxation modulus was registered as a function of time in different temperature ranges depending on the observed *T*_g_ of each CAN. In all cases, full stress relaxation was accomplished (Figure 2 and S17–S21). Due to the complete relaxation of all CANs following a Maxwell model, it was feasible to adjust to the Arrhenius law using relaxation times at τ* = 1/e, by plotting ln (τ*) vs 1000/T and therefore it is possible to calculate the activation energy (*E*_a_) (SI for calculation details). The *E*_a_ of the CANs ranges from 39.6 to 95.9 kJ mol^–1^ (Figure 2 and S17–S21), values that are in good agreement with other polyimine-based CANs previously reported (12.3–129 kJ/mol).^23,24,35^ The observed ranges of *E*_a_ followed an inverted trend with the υ_e_ obtained by DMA. This phenomenon is explained by the increase of mobility of the polymer chains upon the reduction of the cross-link density, and hence, regardless of the **Di-Van** functionality, the designed CANs with higher amount of the flexible long chain fatty acid-based amine to the short triamine portrayed lower *E*_a_. Also, for the CANs with a **Di-Van** bearing an allyloxy functionality, the *E*_a_ was lower than the methoxy-based CANs (Table 1, entries 1, 2, 4, and 5). This is related to the higher flexibility of the allyloxy-based CANs compared to the methoxy-based CANs, which ultimately facilitates the reaction exchange. The *E*_a_ has been proven to be affected by a large number of factors like the chemical structure of reactants in terms of molecular weight, number of reactive functional groups within the reagents, stoichiometry of the reagents involved in the formation of the CAN, etc.^24^ Therefore, the inverted trend of the *E*_a_ with respect the υ_e_ can be used as an argument to justify the observed values in the case of the **Di-Van-OAllyl**-based CANs (Table 1, entries 4 and 5) where **AllylO-Pri**_**1**_**-TAEA**_**0**_ displayed a nonexpected higher υ_e_ (202 ± 13 mol m^–3^) compared to **AllylO-Pri**_**0.66**_**-TAEA**_**0.33**_ (168 ± 41 mol m^–3^) and counterintuitive *E*_a_ values of 46.9 and 72.6 kJ mol^–1^, respectively. In addition, it was also observed that the “most” highly cross-linked CANs were according only to the molecular weight of the amines tested, *i.e*., **MeO-Pri**_**0.33**_**-TAEA**_**0.66**_ and **AllylO-Pri**_**0.33**_**-TAEA**_**0.66**_ displayed the lowest *E*_a_ (49.1 and 39.6 kJ mol^–1^, respectively) compared to the less highly cross-linked CANs attending merely to the molecular weight of the tested amines (Table 1, entries 3 and 6). All in all, as we aimed to tailor the thermal and mechanical properties of the CANs, more than one parameter known to have an impact on the *E*_a_ of the imine-based CANs were altered simultaneously and therefore nonexpected trends in the *E*_a_ have been observed. Leibler, a pioneer in CAN research, and co-workers introduced an unique feature of CANs know as topology freezing transition temperature (*T*_v_).^55^ This temperature provides information about the service temperature of the material, as it is considered that above this temperature, exchange reactions take place and the material can easily be reprocessed and recycled. On the other hand, below the aforementioned temperature, exchange reactions are slow and therefore the CAN displays a more classical thermoset behavior. For this purpose, frequency sweep experiments were performed on all CANs at room temperature (Figures S22–S27). It was then possible to use the Maxwell equation obtained from stress-relaxation experiments to calculate *T*_v_ (SI for calculation details) (Table 1). The obtained values agree with the observed trends for a CAN-like material in which *T*_g_ > *T*_v_ following a William–Landel–Ferry behavior.^56^ In addition, it was observed that the service window of the networks moved accordingly to their Δ(*T*_g_ – *T*_v_) without significant differences. In this scenario, it is proposed that the likelihood of the whole network to rearrange is rather high due to the low *E*_a_ of the exchange reaction. However, it is also noteworthy that the obtained *T*_v_ values must be interpreted as a theoretical value that is extrapolated from the stress-relaxation plots in agreement with the Arrhenius law and Maxwell equation, and therefore *T*_v_ has no actual physical meaning, as it is claimed that the kinetics of the molecular rearrangements within the network are strongly hindered by the lack of free volume and frozen segmental motion.^57^

**Figure 3:**
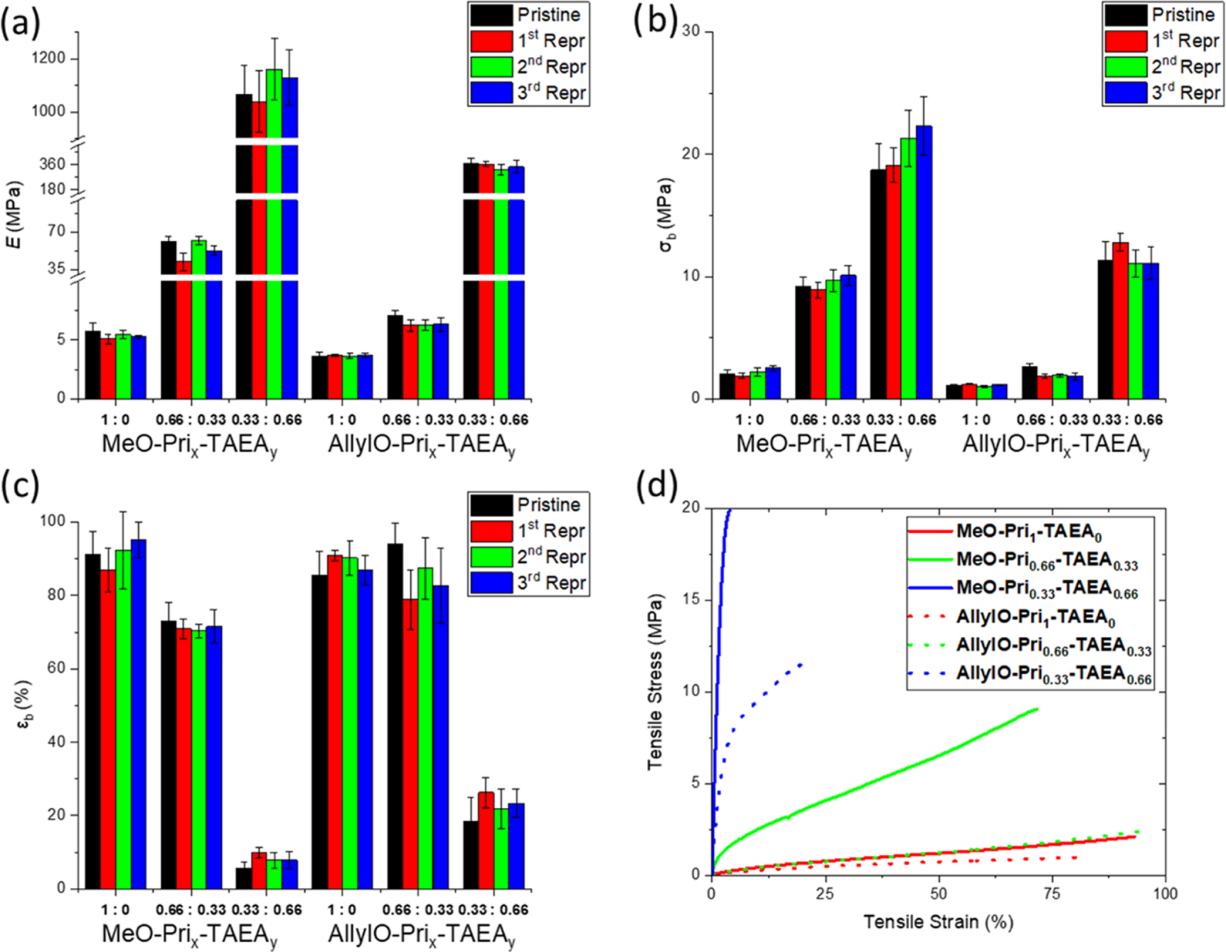
Young′s modulus (*E*) (*left top*, a), tensile strength at break (σ_b_) (*right top*, b), and elongation at break (ε_b_) (*left bottom*, c) of the pristine, first, second, and third reprocessing of CANs **MeO-Pri**_**x**_**-TAEA**_**y**_ and **AllylO-Pri**_**x**_**-TAEA**_**y**_ after hot-pressing at 140 °C for 20 min. Stress–strain curves of CANs **MeO-Pri**_**x**_**-TAEA**_**y**_ and **AllylO-Pri**_**x**_**-TAEA**_**y**_ (*right bottom*, d).

The mechanical properties of the biobased CANs were evaluated and shown to portray a large range of properties adaptable by the choice of **Di-Van** core functionality as well as the nature and composition of the di/triamine (Figure 3). First, in order to prepare suitable specimens for which the mechanical properties could be evaluated, cured CANs were ground, followed by hot press processing at the same temperature that they were cured, *i.e*., 140 °C for 20 min. This process would be repeated up to three times to demonstrate the reprocessability of the cross-linked materials (Figures S28–S33). Two different trends were observed depending both on the nature of the **Di-Van** functionality and also the varying ratios of the cross-linker amines. Here, the CANs based on monomer **Di-Van-OMe** bearing methoxy groups in all cases displayed higher Young modulus (*E*) and strength at break (σ_b_), where **MeO-Pri**_**0.66**_**-TAEA**_**0.33**_ was the toughest one, while **MeO-Pri**_**1**_**-TAEA**_**0**_ resulted to be the most brittle behavior. This correlates with the higher flexibility of the allyloxy moieties when compared with methoxy ones. Still, the variation in the ratios of cross-linker had a much more dramatic impact on the mechanical properties. CANs fully composed of flexible long chain fatty acid-based amine **MeO-Pri**_**1**_**-TAEA**_**0**_ and **AllylO-Pri**_**1**_**-TAEA**_**0**_ exhibited high elongation values ε_b_ = 91.1 ± 6.4 and 85.5 ± 6.6%, respectively, whereas both values of *E* = 5.76 ± 0.64 and 3.61 ± 0.34 MPa and σ_b_ = 2.06 ± 0.28 and 1.07 ± 0.13 MPa were low denoting the softness of the obtained CANs (Table S1, entries 1–4 and 13–16). The incorporation of a short triamine like TAEA resulted in CANs with a higher cross-linking density, as previously corroborated by DMA analysis, which still led to elastic materials **MeO-Pri**_**0.66**_**-TAEA**_**0.33**_ and **AllylO-Pri**_**0.66**_**-TAEA**_**0.33**_ with elongation values of ε_b_ = 73.1 ± 4.9 and 94.0 ± 5.8%, respectively (Table S1, entries 5 and 17). Despite the loss of elasticity in the case of CANs **MeO-Pri**_**0.66**_**-TAEA**_**0.33**_ derived from monomer **Di-Van-OMe** when compared with **MeO-Pri**_**1**_**-TAEA**_**0**_, a significant increase of both *E* and σ_b_ was observed from 5.76 ± 0.64 to 65.4 ± 8.9 MPa and from 2.06 ± 0.28 to 9.2 ± 0.8 MPa, respectively (Table S1, entries 1 and 5). In this regard, the increase in the mechanical properties was not as significant for the allyloxy-modified divanillin CANs compared to the methoxy-based divanillin CANs, again indicating the importance of not only the amine-combination but also the functionality of divanillin (Table S1, entries 13 and 17). Finally, when higher ratios of **TAEA** were employed, the CANs were much stiffer, with an elongation at break decreased to 5.7 ± 1.6 and 18.3 ± 6.7%, respectively (Table S1, entries 9 and 21). *E* and σ_b_ showed an extraordinary increase in both cases being higher than that for **MeO-Pri**_**0.33**_**-TAEA**_**0.66**_ bearing a methoxy group in its structure, which brings a much higher rigidity than the allyloxy-based CANs (Table S1, entries 9 and 13–24). These mechanical properties were compared with other vanillin-based imine CANs which used at least in one of the tested amines in this work. For example, imine-based CANs containing a vanillin moiety linked via the phenol functionality to either a furfuryl or succinyl ester reacted with equimolar amounts of fatty acid-based amine and exhibited a three times higher elongation in the furfuryl-based CAN (340 ± 14% vs 91.1 ± 6.4% and 85.5 ± 6.6%) and with slightly higher σ_b_ values (2.45 ± 0.08 MPa vs 2.06 ± 0.28 MPa and 1.07 ± 0.28 MPa), nevertheless, when compared with the succinyl-based one (ε_b_ = 17.9 ± 0.8% and σ_b_ = 1.04 ± 0.02 MPa) both methoxy and allyloxy displayed higher values.

**Figure 4:**
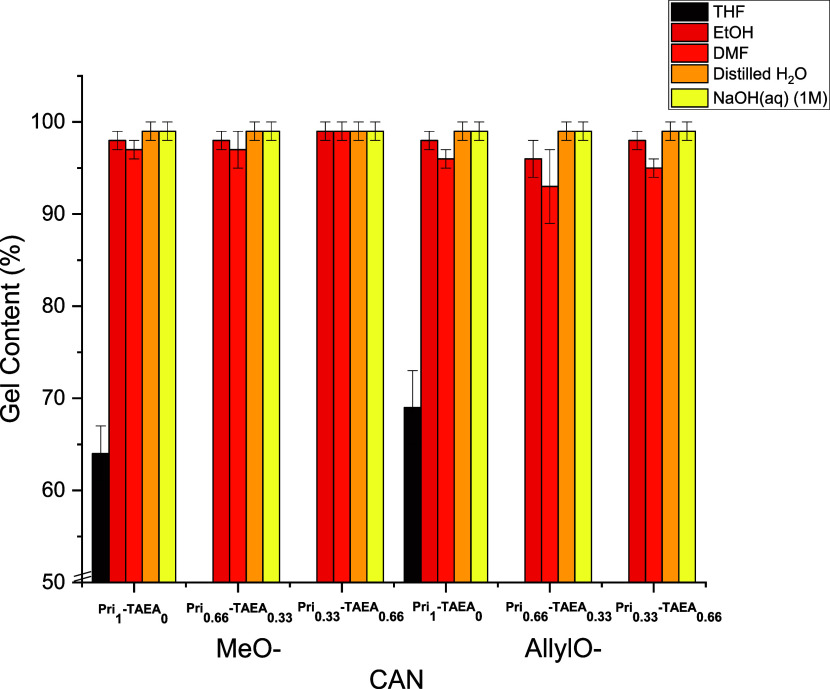
Gel contents after immersion in different media for 24 h at r.t of CANs **MeO-Pri**_**x**_**-TAEA**_**y**_ and **AllylO-Pri**_**x**_**-TAEA**_**y**_.

One of the characteristic features of thermosets, among others, is their chemical stability toward different solvents. This is a result of their inherent irreversible cross-linked nature. Yet, the presence of dynamic reversible chemistries present in CANs allows the possibility of chemical recycling as a consequence of the reversibility present in the network while keeping the chemical resistance toward different solvents. All CANs showed a high chemical stability in polar solvents such as EtOH and DMF having gel contents >95% in all cases (Figure 4). CANs containing **TAEA** in their network structure quickly swelled prior to becoming fully soluble when immersed in THF. For the CANs with only fatty acid-based amine, a gel content of 64 and 69% was obtained for both **Di-Van** CANs. The CANs also withstood at pH = 7 and under basic conditions when immersed in a solution of NaOH (1 M). This high stability toward hydrolysis can be explained by the highly hydrophobic nature of the CANs itself due to the presence of aromatic moieties from the vanillin core along with the presence of fatty acid-based amine, which is comparable to other polyimine-based CANs bearing the fatty acid-based amine.^58^

Thanks to the inherent nature of the imine-bonds’ sensitivity to acidic conditions, chemical recycling is possible, and when the CANs were immersed in an aqueous solution of HCl (1 M), the network lost their integrity and a solid residue was obtained. The imine functional group is basic, and under acidic conditions, it can be protonated and transformed into iminium ions, which are more susceptible to be attacked by nucleophiles, in this case water, rendering both the starting aldehyde and amines. The aforementioned residue, using **MeO-Pri**_**0.66**_**-TAEA**_**0.33**_ as an example, was dissolved in CDCl_3_, and a ^1^H NMR spectrum was recorded (Figure S34). This confirmed hydrolysis of the imine as the peak corresponding to the aldehyde moiety was observed at 9.91 ppm and no peak associated with the imine was observed at lower chemical shifts around 8.00–8.50 ppm in ^1^H NMR.

**Figure 5:**
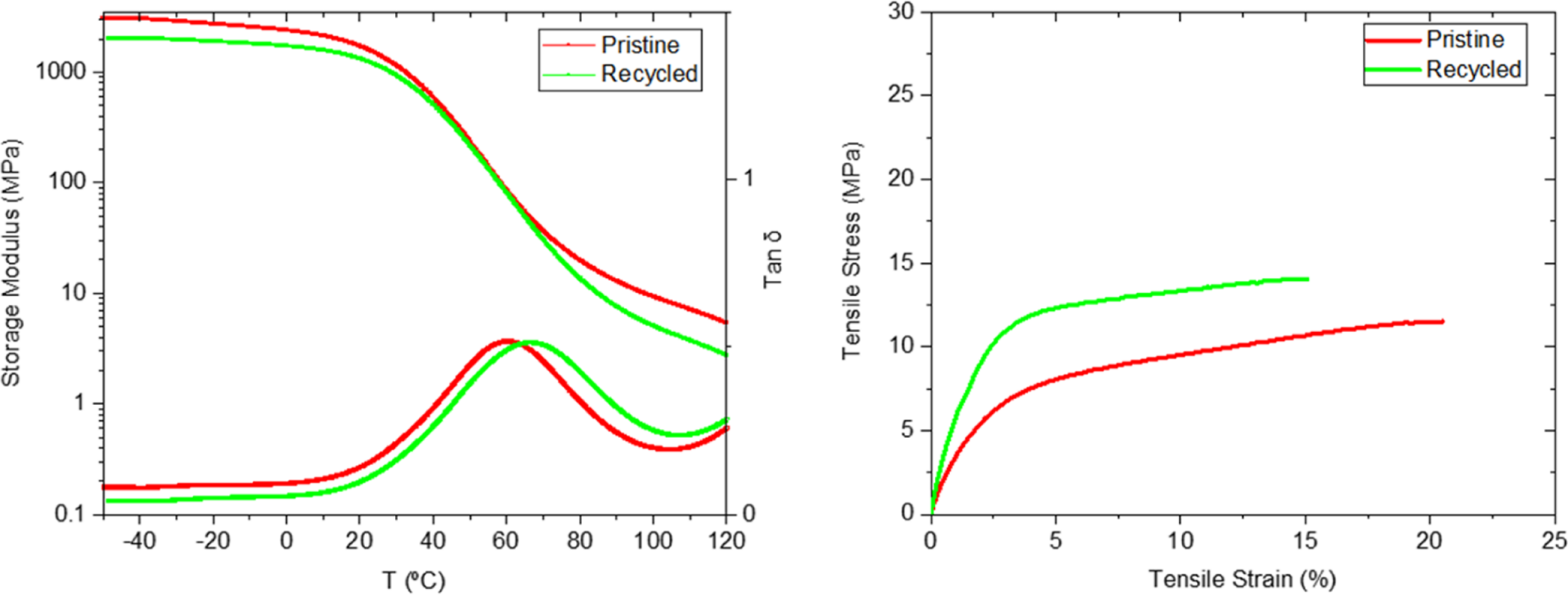
DMA curve of pristine and chemically recycled CAN **AllylO-Pri**_**0.33**_**-TAEA**_**0.66**_ (*left*) and stress–strain curve of pristine and chemically recycled CAN **AllylO-Pri**_**0.33**_**-TAEA**_**0.66**_ (*right*).

The reversibility of the imine functionality under acidic conditions allows for chemical recycling of imine-based CANs. As a model CAN, we chose **AllylO-Pri**_**0.33**_**-TAEA**_**0.66**_ utilizing **Di-Van-OAllyl** (2 g, 5.24 mmol, 1 equiv), **TAEA** (0.52 mL, 3.46 mmol, 0.66 equiv), and **Pri** (1.07 g, 1.73 mmol, 0.33 equiv). The obtained CAN was cut into small pieces and suspended in a 0.1 M solution of HCl (500 mL) overnight at room temperature. The film was degraded and a thin yellowish powder, was precipitated and filtered over a Büchner funnel and dried under high vacuum at 60 °C. The monomer **Di-Van-OAllyl** (1.30 g, 3.41 mmol) was recovered with a 65% yield and further confirmation that an isolated solid was solely the desired divanillin monomer achieved by ^1^H NMR spectroscopy (Figure S35). The so-recovered monomer was recast and later cured using the same molar ratios that the original CAN yielded, yielding a chemically recycled **AllylO-Pri**_**0.33**_**-TAEA**_**0.66**_. Similarly, to previous depicted examples, formation of the CAN was confirmed by FTIR spectroscopy performed before and after the curing process (Figure S36). In pursuit of evaluating the efficiency of the chemical recycling and the subsequent film recasting, DMA analysis along with a uniaxial tensile test was performed (Figure 5). When compared, the chemically recycled CAN exhibited a higher *T*_g_ (66 °C) compared to the pristine CAN (61 °C). The mechanical testing showed that the recycled material exhibited a slightly less elastic behavior with a slight decrease in the elongation at break value from ε_b_ = 18.8 ± 6.8% to 16.1 ± 3.0, while both *E* and σ_b_ increased from 372 ± 32 to 730 ± 38 MPa and from 11.3 ± 1.6 to 13.8 ± 1.2 MPa, respectively.

## Conclusions

A series of biobased polyimine CANs were prepared using a combination of divanillin-functionalized monomers with a short triamine and a flexible fatty acid dimer–trimer long chain amine. Viscoelastic properties were tailored by tuning the ratios of short and long polyamines leading to a range of *T*_g_ values from 16 to 61 °C. It was observed that the tailoring of the phenol functionality of the aromatic core with moieties with different flexibilities such as methoxy and allyloxy groups did not exhibit smaller effects compared to the alterations of the ratios of the amines employed. The mechanical properties were also highly influenced by the ratios of the two different amines employed in this work. When higher contents of short amine, **TAEA**, was utilized, more rigid highly cross-linked CANs were obtained displaying lower elasticity with high *E* and σ_b_ values. On the contrary, when the ratio of flexible fatty acid-based amine was higher, the CANs exhibited high elasticity with elongation values (ε_b_) up to 94%. The CANs were able to be reprocessed up to 3 times without jeopardizing their mechanical properties. The reversibility of the imine functionalities present in the obtained networks was successfully demonstrated by stress-relaxation experiments. Chemical recycling was performed via acidic hydrolysis and the divanillin was recovered with good yields. Film recasting was performed, and the thermomechanical properties were compared to the pristine CAN, portraying similar performance.

## Supporting Information Available

NMR characterization of monomers, FTIR analysis, TGA curves, stress-relaxation experiments, frequency sweep experiments, stress–strain curves, calculations for *E*_a_ and *T*_v_, and characterization of the recycled CAN (PDF)
