# Supplementary material for: Covalent Adaptable Networks with Tailorable Material Properties Based on Divanillin Polyimines
Source: Biomacromolecules. 2024 Mar 18;25(4):2348–57. doi: 10.1021/acs.biomac.3c01224 (PMC11005045; doi:10.1021/acs.biomac.3c01224)
Supplement: Supplementary file 1 — bm3c01224_si_001.pdf [file bm3c01224_si_001.pdf]

Covalent Adaptable Networks with Tailorable Material Properties Based on Divanillin Polyimines

Noé Fanjul-Mosteirín,[1] and Karin Odelius[1]*

[1]Wallenberg Wood Science Center, WWSC, Department of Fibre and Polymer Technology, KTH Royal Institute of Technology, SE-100 44, Stockholm, Sweden

*Corresponding author: Karin Odelius, hoem@kth.se

Table of Contents

^{1}H and ^{13}C NMR spectra **3**

Figure S1. ^{1}H NMR spectrum of **Di-Van** (400 MHz, 298K, DMSO). 3

Figure S2. ^{13}C APT NMR spectrum of **Di-Van** (100 MHz, 298 K, DMSO). 3

Figure S3. ^{1}H NMR spectrum of **Di-Van-OMe** (400 MHz, 298K, DMSO). 4

Figure S4. ^{13}C APT NMR spectrum of **Di-Van-OMe** (100 MHz, 298 K, DMSO). 4

Figure S5. ^{1}H NMR spectrum of **Di-Van-OAllyl** (400 MHz, 298K, $CDCl_3$). 5

Figure S6. ^{13}C APT NMR spectrum of **Di-Van-OAllyl** (100 MHz, 298 K, $CDCl_3$). 5

Figure S7. ^{1}H NMR spectrum of octadecylamine-based imine (400 MHz, 298 K, $CDCl_3$). 6

FTIR **7**

Figure S8. FTIR spectrum of monomer **Di-Van-OMe**. 7

Figure S9. FTIR spectrum of monomer **Di-Van-OAllyl**. 7

Figure S10. FTIR spectrum of network **MeO-Pri$_1$-TAEA$_0$** before curing process (*top*), FTIR spectrum of network **MeO-Pri$_1$-TAEA$_0$** after curing process 140 °C during 8 h (*bottom*). 8

Figure S11. FTIR spectrum of network **MeO-Pri$_{0.66}$-TAEA$_{0.33}$** before curing process (*top*), FTIR spectrum of network **MeO-Pri$_{0.66}$-TAEA$_{0.33}$** after curing process 140 °C during 8 h (*bottom*). 9

Figure S12. FTIR spectrum of network **MeO-Pri$_{0.33}$-TAEA$_{0.66}$** before curing process (*top*), FTIR spectrum of network **MeO-Pri$_{0.33}$-TAEA$_{0.66}$** after curing process 140 °C during 8 h (*bottom*). 10

Figure S13. FTIR spectrum of network **AllylO-Pri$_1$-TAEA$_0$** before curing process (*top*), FTIR spectrum of network **AllylO-Pri$_1$-TAEA$_0$** after curing process 140 °C during 8 h (*bottom*). 11

Figure S14. FTIR spectrum of network **AllylO-Pri$_{0.66}$-TAEA$_{0.33}$** before curing process (*top*), FTIR spectrum of network **AllylO-Pri$_{0.66}$-TAEA$_{0.33}$** after curing process 140 °C during 8 h (*bottom*). ... 12

Figure S15. FTIR spectrum of network **AllylO-Pri$_{0.33}$-TAEA$_{0.66}$** before curing process (*top*), FTIR spectrum of network **AllylO-Pri$_{0.33}$-TAEA$_{0.66}$** after curing process 140 °C during 8 h (*bottom*). ... 13

TGA results **14**

Figure S16. TGA curves of monomers **Di-Van-OMe**, **Di-Van-OAllyl** and CANs **MeO-Pri$_x$-TAEA$_y$**, **AllylO-Pri$_x$-TAEA$_y$**. 14

Stress relaxation experiments and Arrhenius obtained plots **15**

Figure S17. Stress relaxation curve of **MeO-Pri$_1$-TAEA$_0$** (*left*) and Arrhenius plot obtained from the relaxation times τ^* used to calculate the E_a of **MeO-Pri$_1$-TAEA$_0$** (*right*). 15

Figure S18. Stress relaxation curve of **MeO-Pri$_{0.66}$-TAEA$_{0.33}$** (*left*) and Arrhenius plot obtained from the relaxation times τ^* used to calculate the E_a of **MeO-Pri$_{0.66}$-TAEA$_{0.33}$** (*right*). 15

Figure S19. Stress relaxation curve of **MeO-Pri$_{0.33}$-TAEA$_{0.66}$** (*left*) and Arrhenius plot obtained from the relaxation times τ^* used to calculate the E_a of **MeO-Pri$_{0.33}$-TAEA$_{0.66}$** (*right*). 16

Figure S20. Stress relaxation curve of **AllylO-Pri$_1$-TAEA$_0$** (*left*) and Arrhenius plot obtained from the relaxation times τ^* used to calculate the E_a of **AllylO-Pri$_1$-TAEA$_0$** (*right*)..............................16

Figure S21. Stress relaxation curve of **AllylO-Pri$_{0.66}$-TAEA$_{0.33}$** (*left*) and Arrhenius plot obtained from the relaxation times τ^* used to calculate the E_a of **AllylO-Pri$_{0.66}$-TAEA$_{0.33}$** (*right*)................17

Frequency sweep experiments 18

Figure S22. Frequency sweep experiment of CAN **MeO-Pri$_1$-TAEA$_0$** at room temperature.........18

Figure S23. Frequency sweep experiment of CAN **MeO-Pri$_{0.66}$-TAEA$_{0.33}$** at room temperature...18

Figure S24. Frequency sweep experiment of CAN **MeO-Pri$_{0.33}$-TAEA$_{0.33}$** at room temperature...19

Figure S25. Frequency sweep experiment of CAN **AllylO-Pri$_1$-TAEA$_0$** at room temperature. 19

Figure S26. Frequency sweep experiment of CAN **AllylO-Pri$_{0.66}$-TAEA$_{0.33}$** at room temperature.20

Figure S27. Frequency sweep experiment of CAN **AllylO-Pri$_{0.33}$-TAEA$_{0.66}$** at room temperature.20

Stress-Strain curves overlapped with reprocessing cycles 21

Figure S28. Stress-strain curves of CAN **MeO-Pri$_1$-TAEA$_0$**...21

Figure S29. Stress-strain curves of CAN **MeO-Pri$_{0.66}$-TAEA$_{0.33}$**...21

Figure S30. Stress-strain curves of CAN **MeO-Pri$_{0.33}$-TAEA$_{0.66}$**...22

Figure S31. Stress-strain curves of CAN **AllylO-Pri$_1$-TAEA$_0$**. 22

Figure S32. Stress-strain curves of CAN **AllylO-Pri$_{0.66}$-TAEA$_{0.33}$**..23

Figure S33. Stress-strain curves of CAN **AllylO-Pri$_{0.33}$-TAEA$_{0.66}$**..23

^{1}H NMR spectra of hydrolyzed CAN MeO-Pri$_{0.66}$-TAEA$_{0.33}$..24

Figure S34. ^{1}H NMR of Praimine 1071 (*top*) ^{1}H NMR of residue after immersing CAN **MeO-Pri$_{0.66}$-TAEA$_{0.33}$** in a solution of an aqueous solution of HCl (1M) at room temperature for 24 h (*bottom*) (400 MHz, 298K, $CDCl_3$). 24

Calculations for stress relaxation derived from activation energy..25

Calculations for vitrimer temperature (T_v) using Arrhenius equation from stress relaxation 25

Table S1. Mechanical properties of CANs MeO-Pri$_x$-TAEA$_y$ and AllylO-Pri$_x$-TAEA$_y$. 27

Table S2. Gel content after immersion in different media for 24 h at r.t of CANs MeO-Pri$_x$-TAEA$_y$ and AllylO-Pri$_x$-TAEA$_y$. 29

Characterization of recovered CAN AllylO-Pri$_{0.33}$-TAEA$_{0.66}$ 31

Figure S35. ^{1}H NMR of isolated **Di-Van-OAllyl** after acidic hydrolysis of CAN **AllylO-Pri$_{0.33}$-TAEA$_{0.66}$** (400 MHz, 298K, $CDCl_3$)..31

Figure S36. FTIR spectrum of recycled network **AllylO-Pri$_{0.33}$-TAEA$_{0.66}$** before curing process (*top*), FTIR spectrum of recycled network **AllylO-Pri$_{0.33}$-TAEA$_{0.66}$** after curing process 140 °C during 8 h (*bottom*). 32

^{1}H and ^{13}C NMR spectra

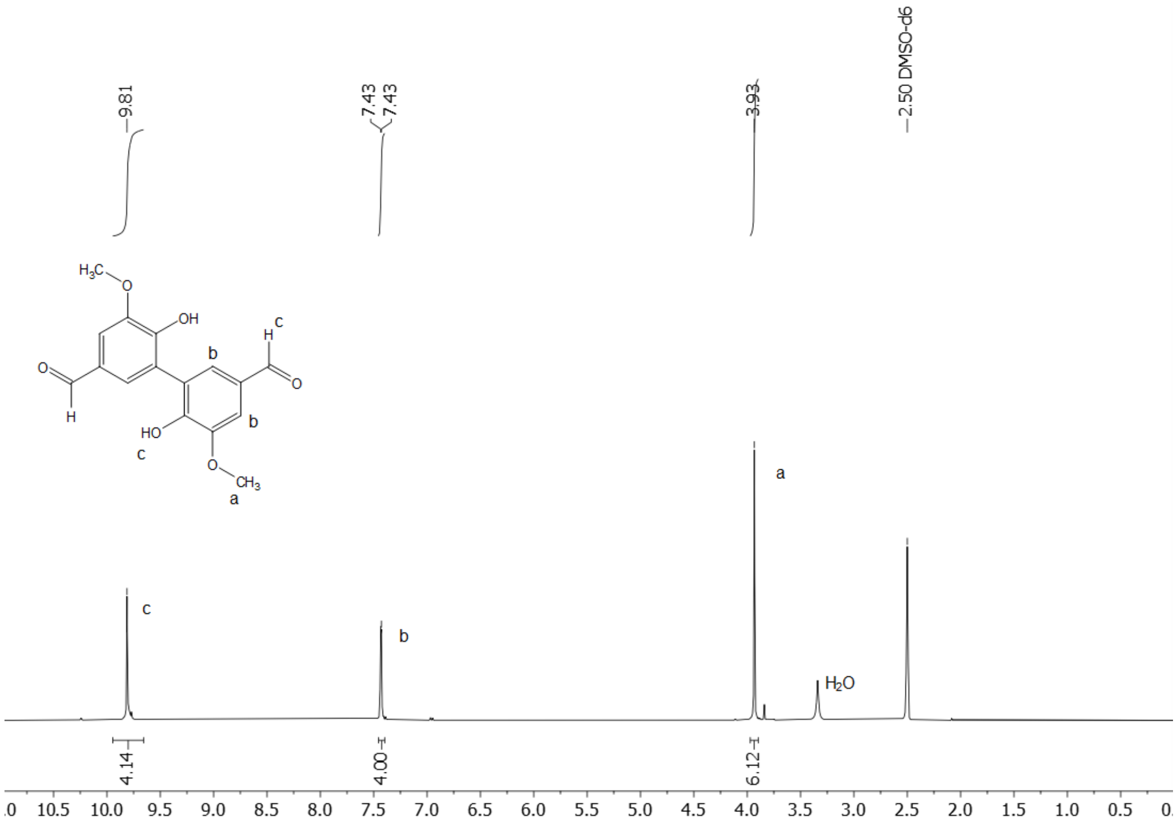

Figure S1. ^{1}H NMR spectrum of **Di-Van** (400 MHz, 298K, DMSO).

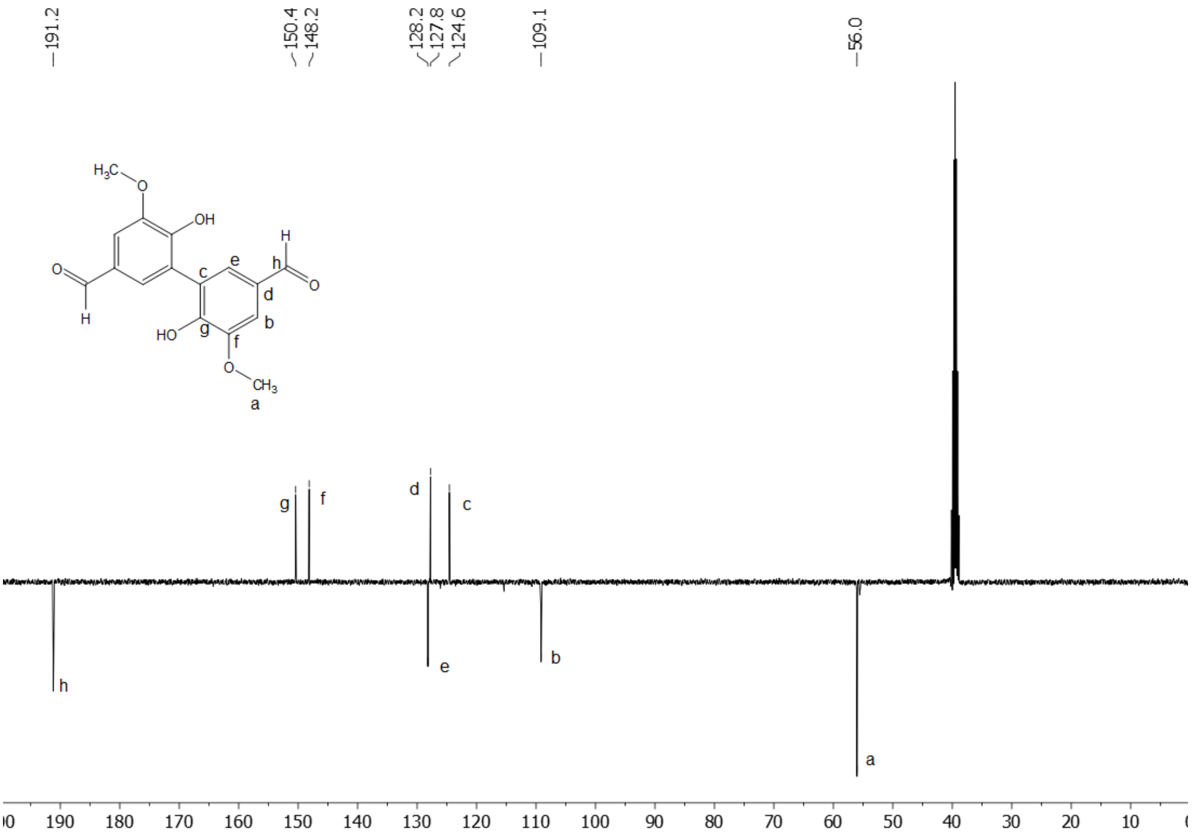

Figure S2. ^{13}C APT NMR spectrum of **Di-Van** (100 MHz, 298 K, DMSO).

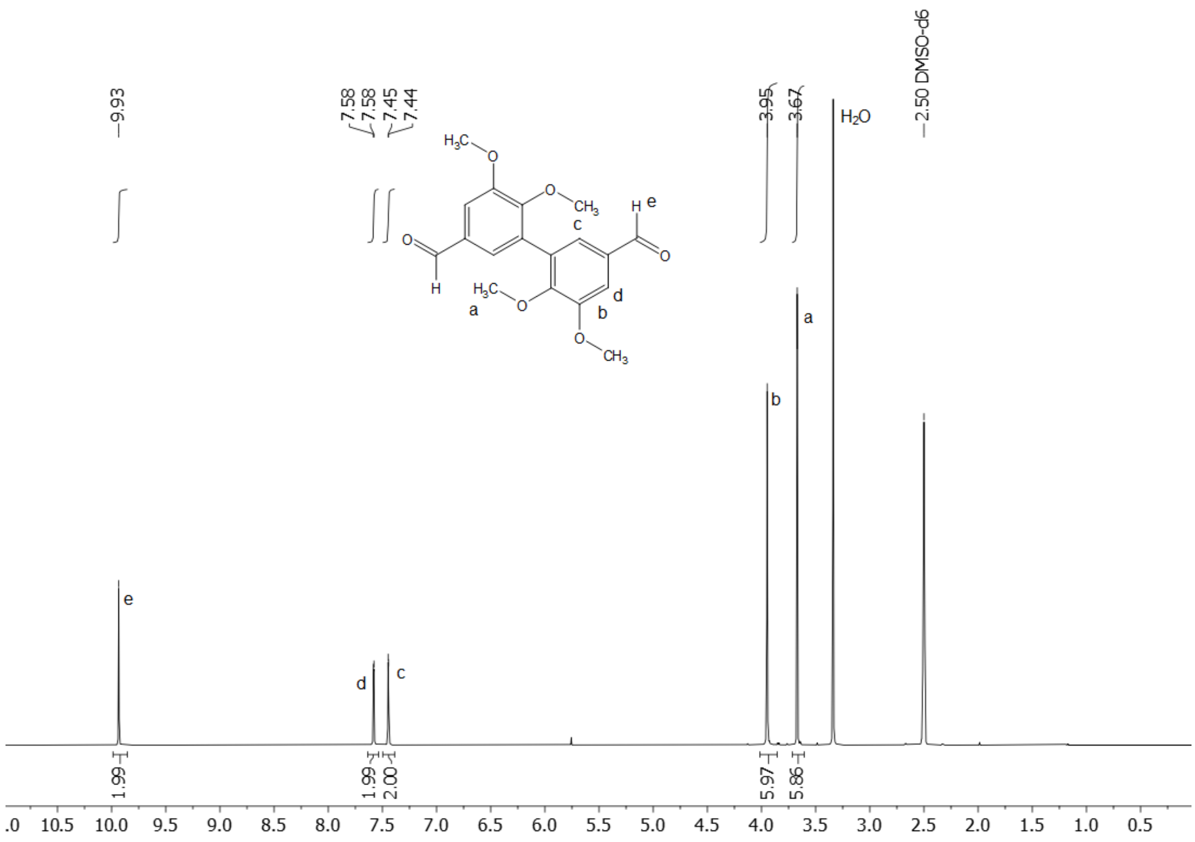

Figure S3. ^{1}H NMR spectrum of **Di-Van-OMe** (400 MHz, 298K, DMSO).

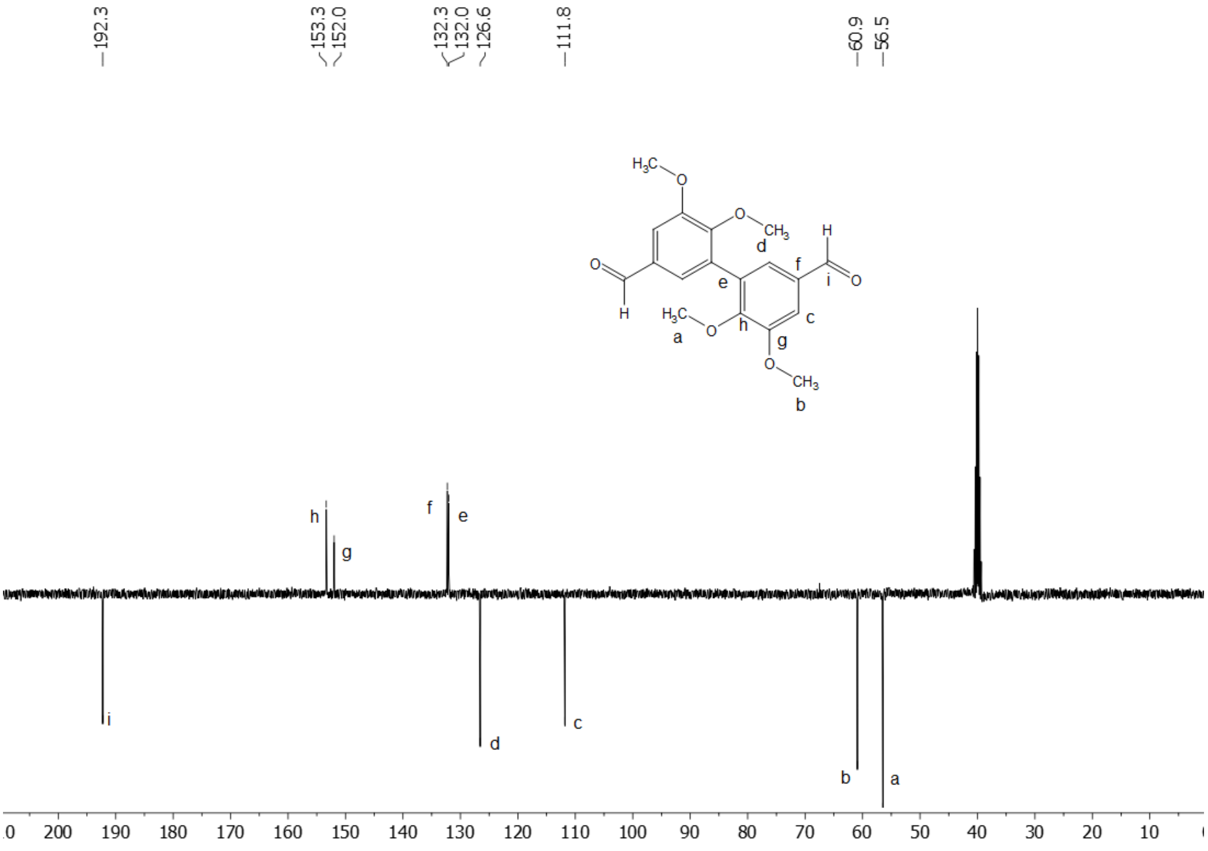

Figure S4. ^{13}C APT NMR spectrum of **Di-Van-OMe** (100 MHz, 298 K, DMSO).

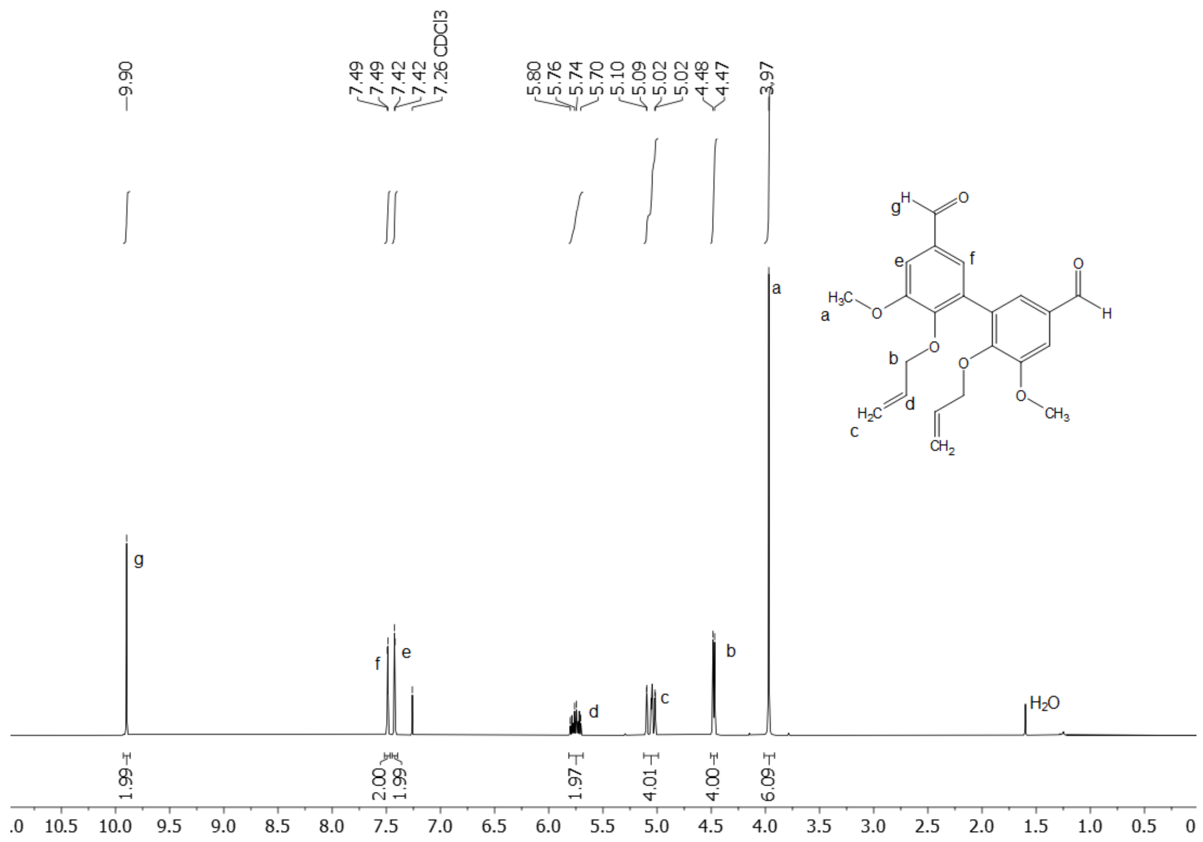

Figure S5. ^{1}H NMR spectrum of **Di-Van-OAllyl** (400 MHz, 298K, $CDCl_3$).

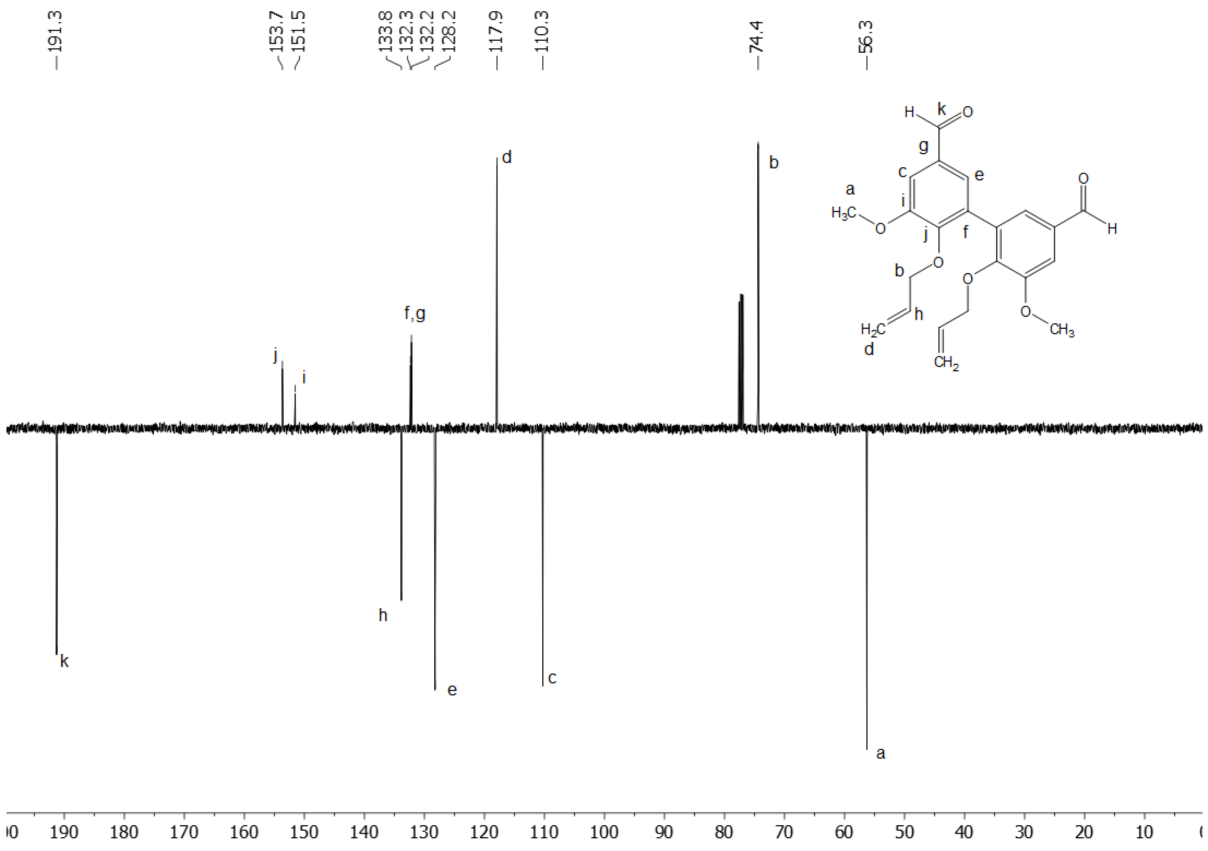

Figure S6. ^{13}C APT NMR spectrum of **Di-Van-OAllyl** (100 MHz, 298 K, $CDCl_3$).

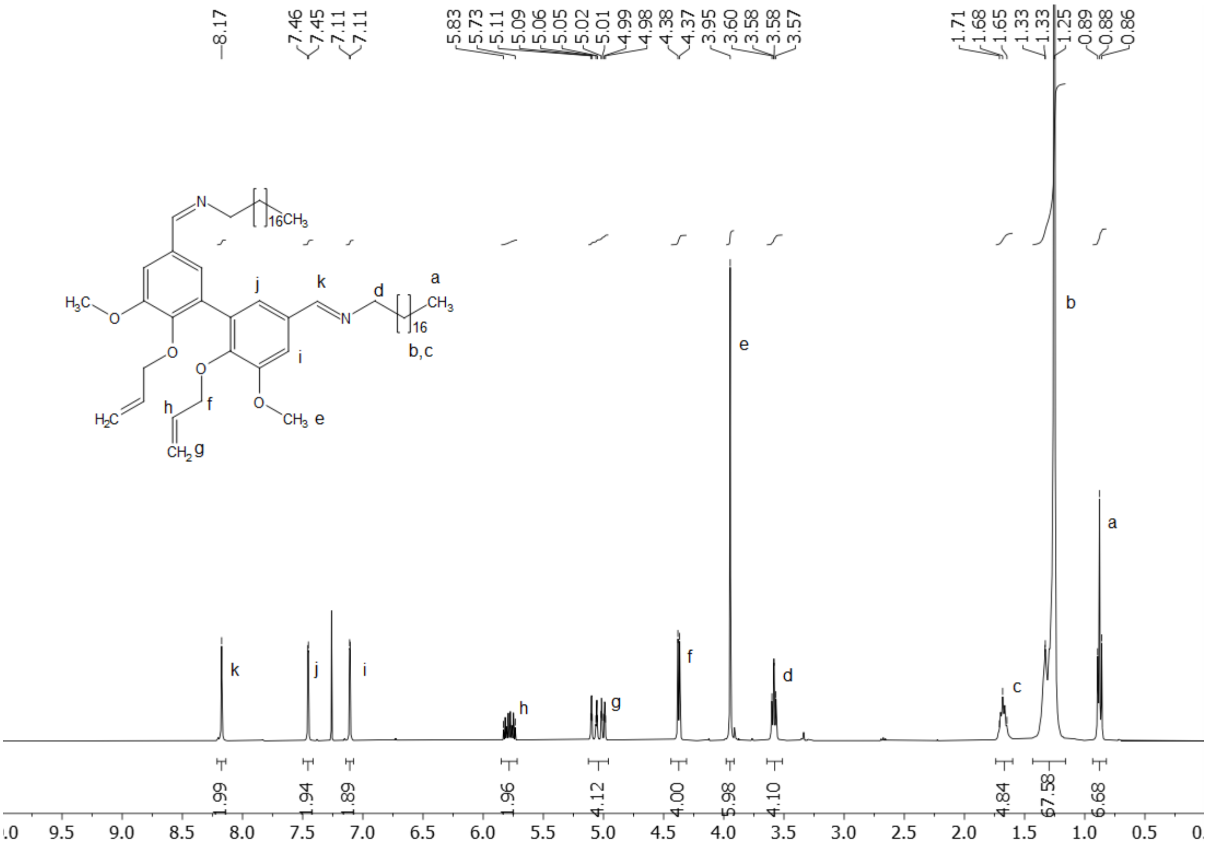

Figure S7. ^{1}H NMR spectrum of octadecylamine-based imine (400 MHz, 298 K, $CDCl_3$).

FTIR

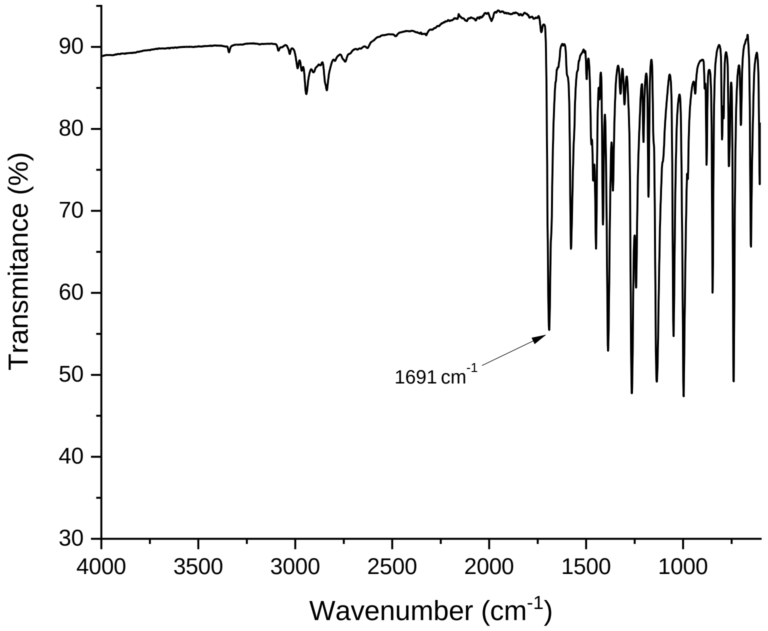

Figure S8. FTIR spectrum of monomer **Di-Van-OMe**.

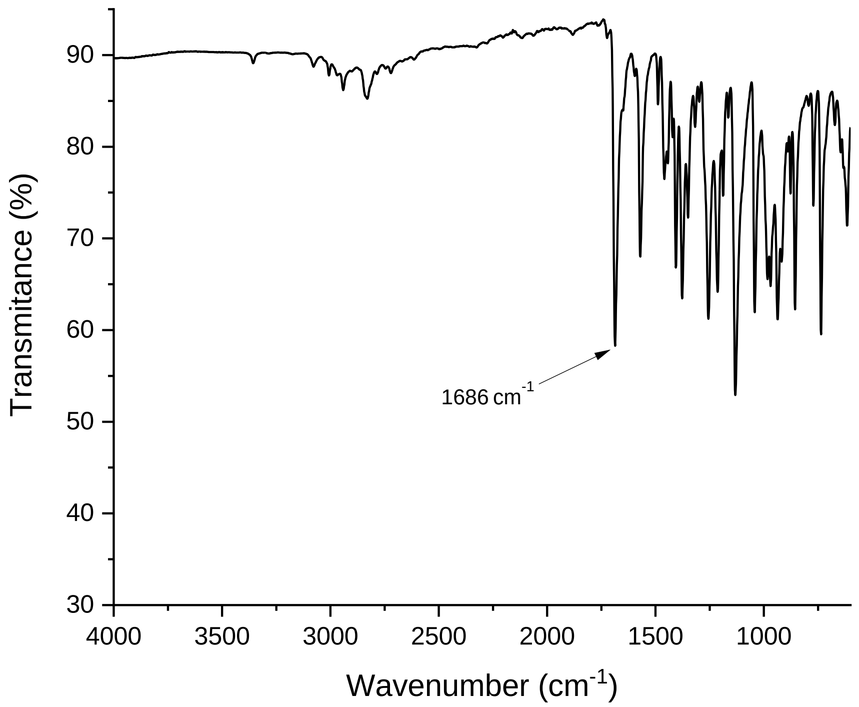

Figure S9. FTIR spectrum of monomer **Di-Van-OAllyl**.

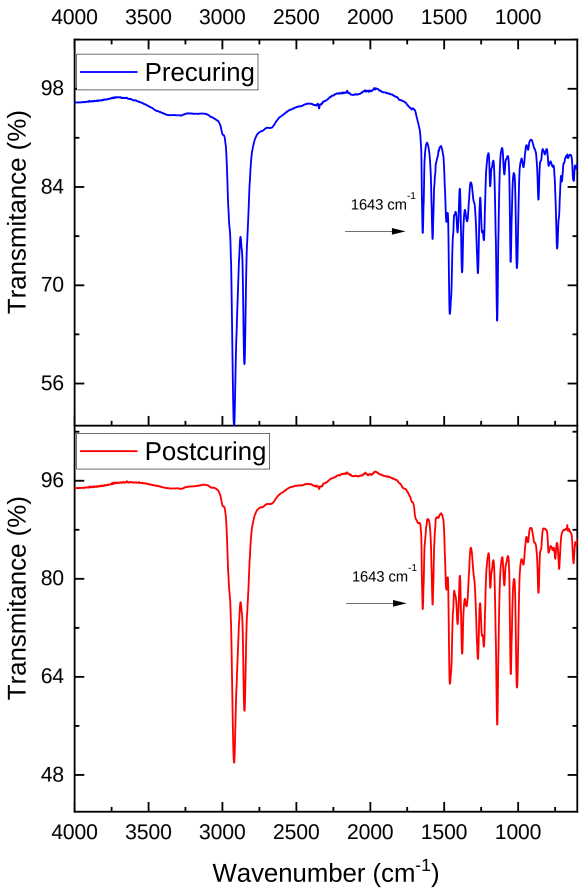

Figure S10. FTIR spectrum of network **MeO-Pri$_1$-TAEA$_0$** before curing process (*top*), FTIR spectrum of network **MeO-Pri$_1$-TAEA$_0$** after curing process 140 °C during 8 h (*bottom*).

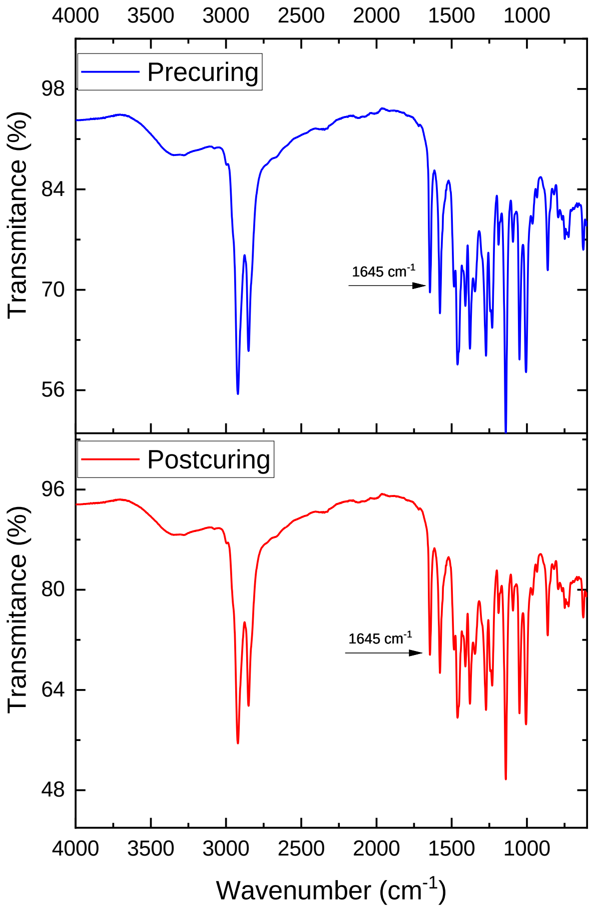

Figure S11. FTIR spectrum of network **MeO-Pri$_{0.66}$-TAEA$_{0.33}$** before curing process (*top*), FTIR spectrum of network **MeO-Pri$_{0.66}$-TAEA$_{0.33}$** after curing process 140 °C during 8 h (*bottom*).

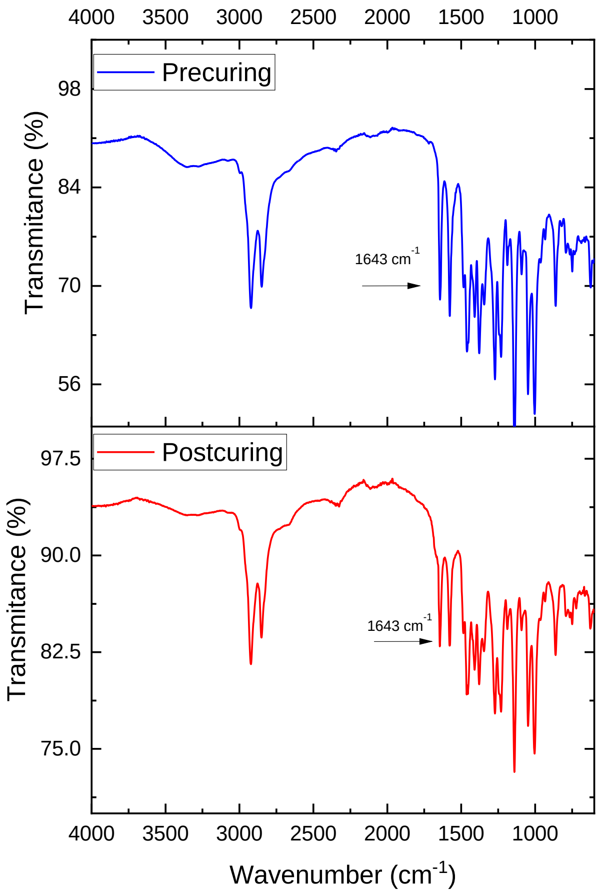

Figure S12. FTIR spectrum of network **MeO-Pri$_{0.33}$-TAEA$_{0.66}$** before curing process (*top*), FTIR spectrum of network **MeO-Pri$_{0.33}$-TAEA$_{0.66}$** after curing process 140 °C during 8 h (*bottom*).

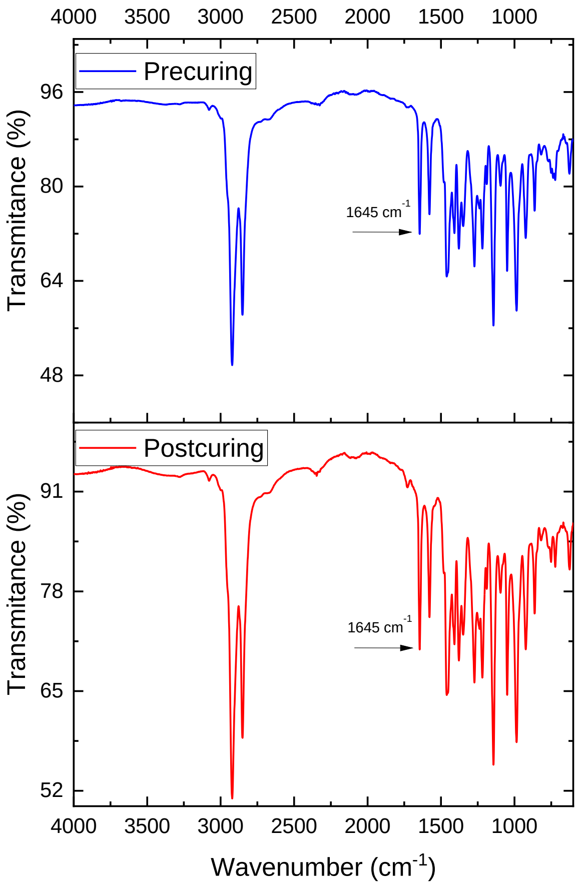

Figure S13. FTIR spectrum of network **AllylO-Pri$_1$-TAEA$_0$** before curing process (*top*), FTIR spectrum of network **AllylO-Pri$_1$-TAEA$_0$** after curing process 140 °C during 8 h (*bottom*).

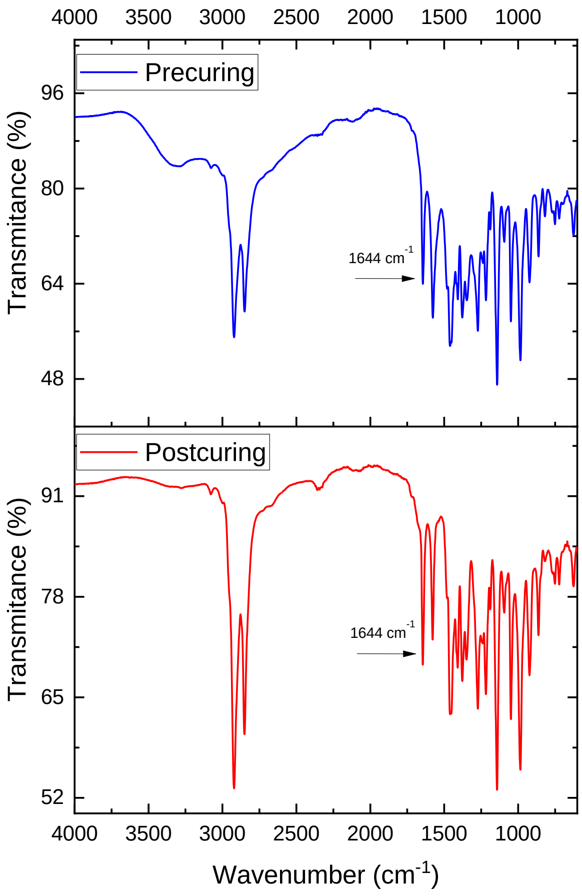

Figure S14. FTIR spectrum of network **AllylO-Pri$_{0.66}$-TAEA$_{0.33}$** before curing process (*top*), FTIR spectrum of network **AllylO-Pri$_{0.66}$-TAEA$_{0.33}$** after curing process 140 °C during 8 h (*bottom*).

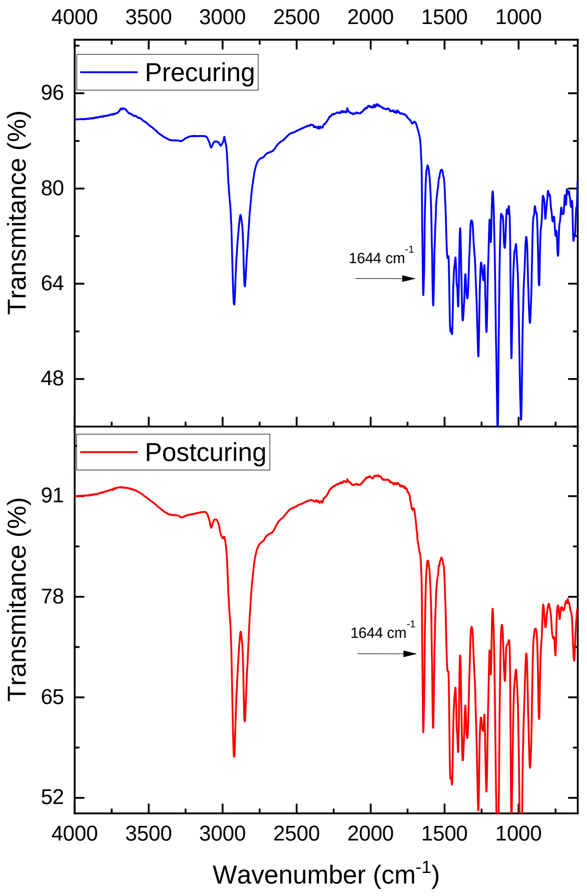

Figure S15. FTIR spectrum of network **AllylO-Pri$_{0.33}$-TAEA$_{0.66}$** before curing process (*top*), FTIR spectrum of network **AllylO-Pri$_{0.33}$-TAEA$_{0.66}$** after curing process 140 °C during 8 h (*bottom*).

TGA results

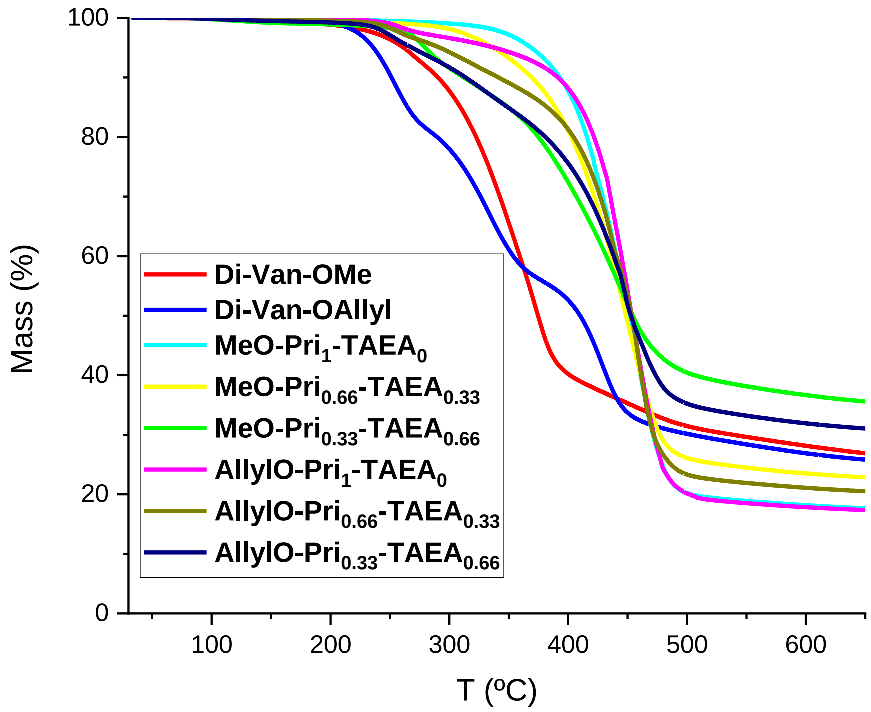

Figure S16. TGA curves of monomers **Di-Van-OMe**, **Di-Van-OAllyl** and CANs **MeO-Pri$_x$-TAEA$_y$**, **AllylO-Pri$_x$-TAEA$_y$**.

Stress relaxation experiments and Arrhenius obtained plots

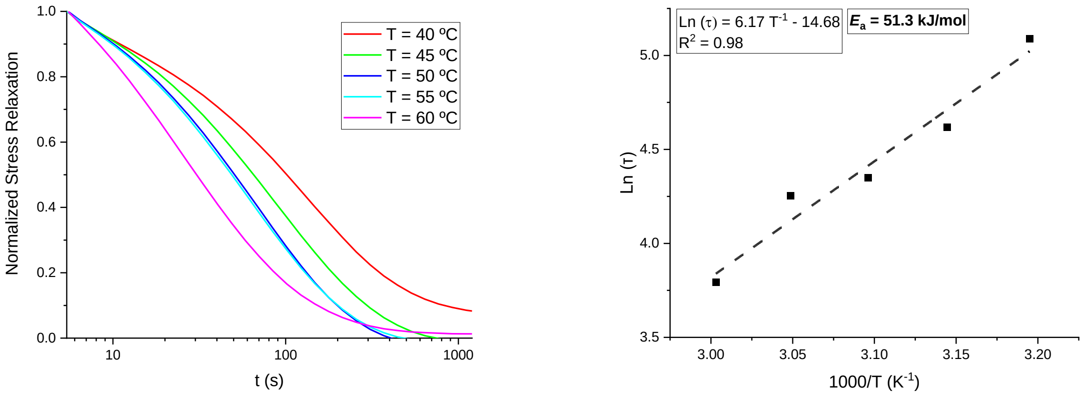

Figure S17. Stress relaxation curve of **MeO-Pri$_1$-TAEA$_0$** (*left*) and Arrhenius plot obtained from the relaxation times τ^* used to calculate the E_a of **MeO-Pri$_1$-TAEA$_0$** (*right*).

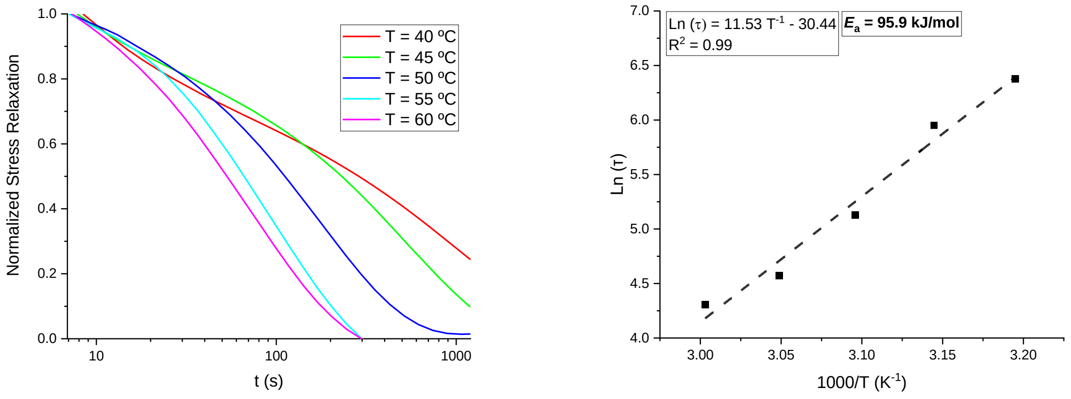

Figure S18. Stress relaxation curve of **MeO-Pri$_{0.66}$-TAEA$_{0.33}$** (*left*) and Arrhenius plot obtained from the relaxation times τ^* used to calculate the E_a of **MeO-Pri$_{0.66}$-TAEA$_{0.33}$** (*right*).

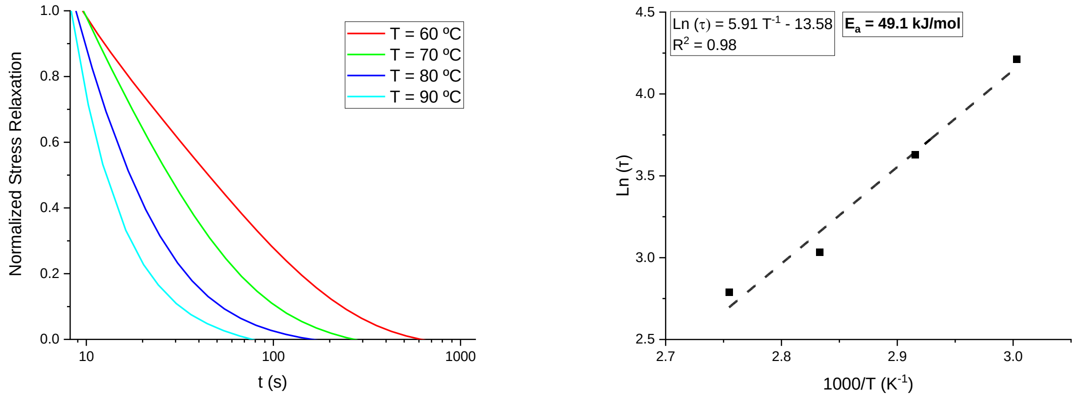

Figure S19. Stress relaxation curve of **MeO-Pri0.33-TAEA0.66** (*left*) and Arrhenius plot obtained from the relaxation times τ^* used to calculate the E_a of **MeO-Pri0.33-TAEA0.66** (*right*).

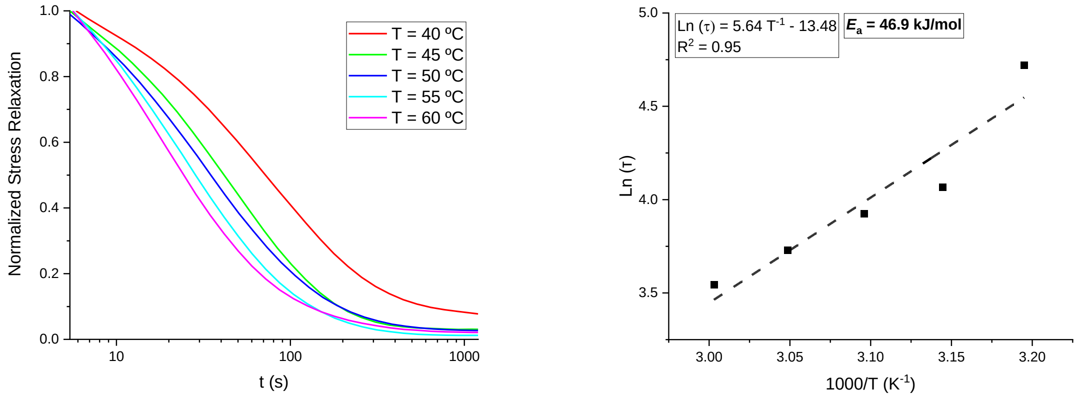

Figure S20. Stress relaxation curve of **AllylO-Pri1-TAEA0** (*left*) and Arrhenius plot obtained from the relaxation times τ^* used to calculate the E_a of **AllylO-Pri1-TAEA0** (*right*).

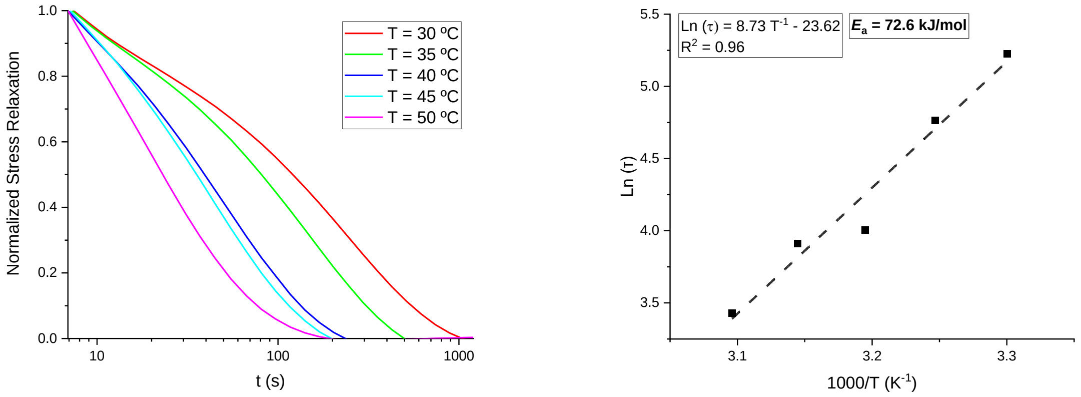

Figure S21. Stress relaxation curve of **AllylO-Pri$_{0.66}$-TAEA$_{0.33}$** (*left*) and Arrhenius plot obtained from the relaxation times τ^* used to calculate the E_a of **AllylO-Pri$_{0.66}$-TAEA$_{0.33}$** (*right*).

Frequency sweep experiments

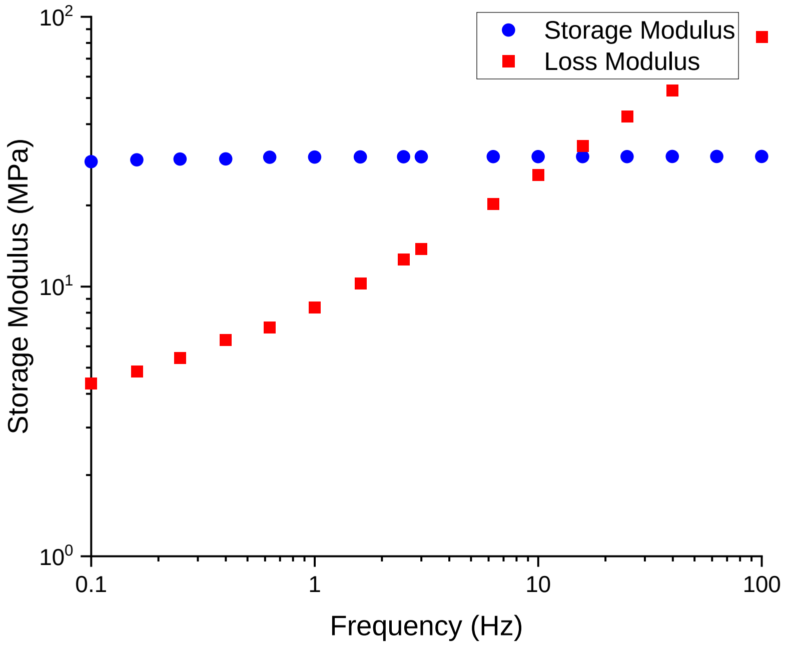

Figure S22. Frequency sweep experiment of CAN **MeO-Pri$_1$-TAEA$_0$** at room temperature.

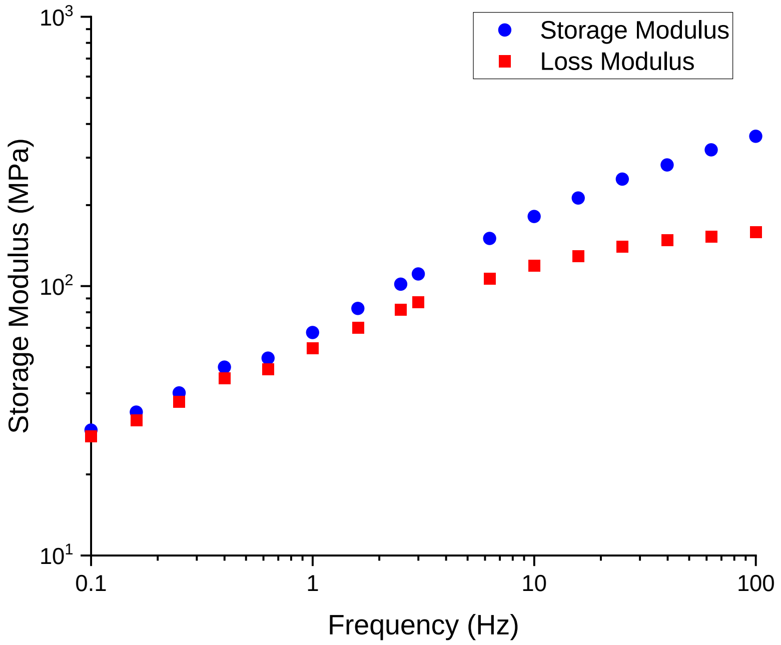

Figure S23. Frequency sweep experiment of CAN **MeO-Pri$_{0.66}$-TAEA$_{0.33}$** at room temperature.

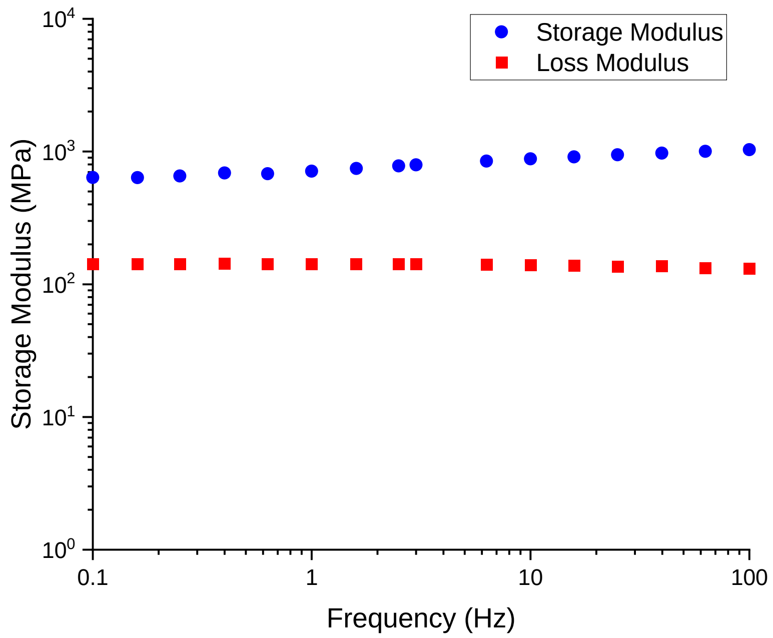

Figure S24. Frequency sweep experiment of CAN **MeO-Pri$_{0.33}$-TAEA$_{0.33}$** at room temperature.

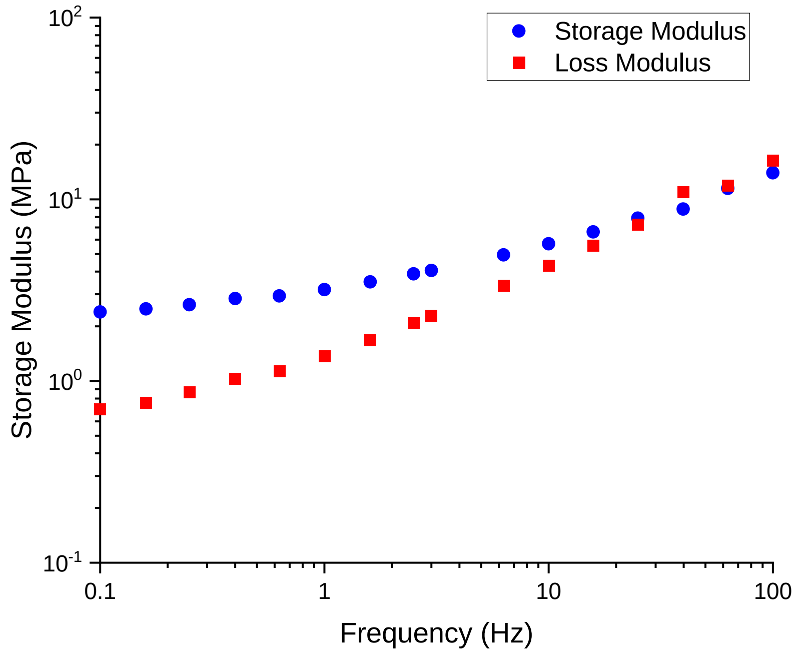

Figure S25. Frequency sweep experiment of CAN **AllylO-Pri$_1$-TAEA$_0$** at room temperature.

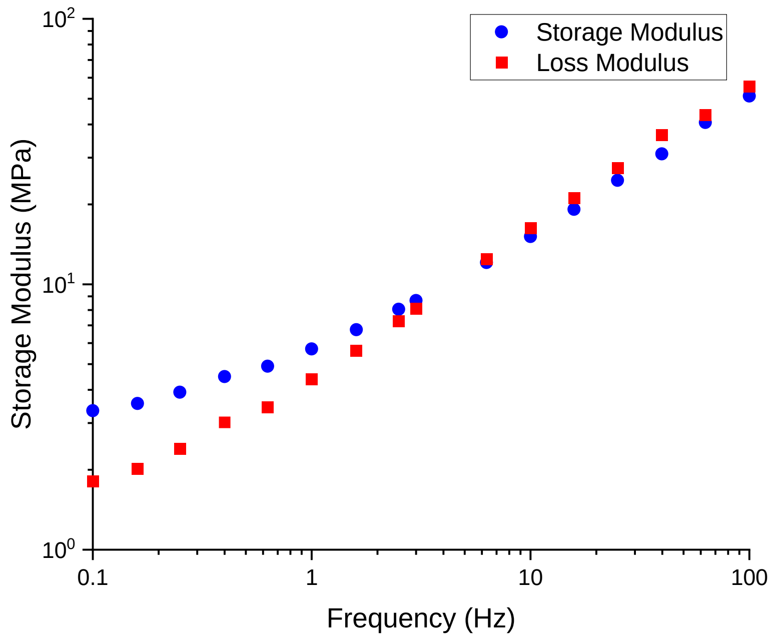

Figure S26. Frequency sweep experiment of CAN **AllylO-Pri$_{0.66}$-TAEA$_{0.33}$** at room temperature.

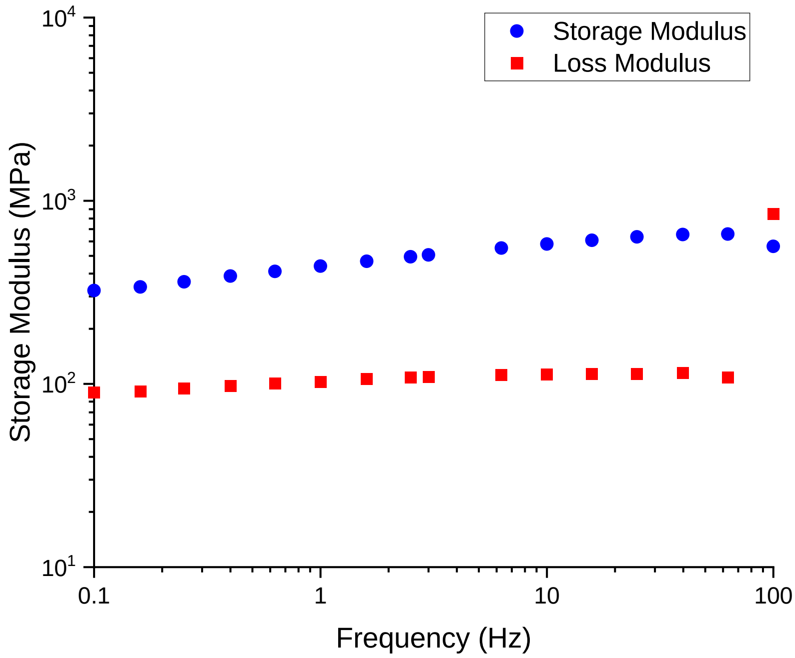

Figure S27. Frequency sweep experiment of CAN **AllylO-Pri$_{0.33}$-TAEA$_{0.66}$** at room temperature.

Stress-Strain curves overlapped with reprocessing cycles

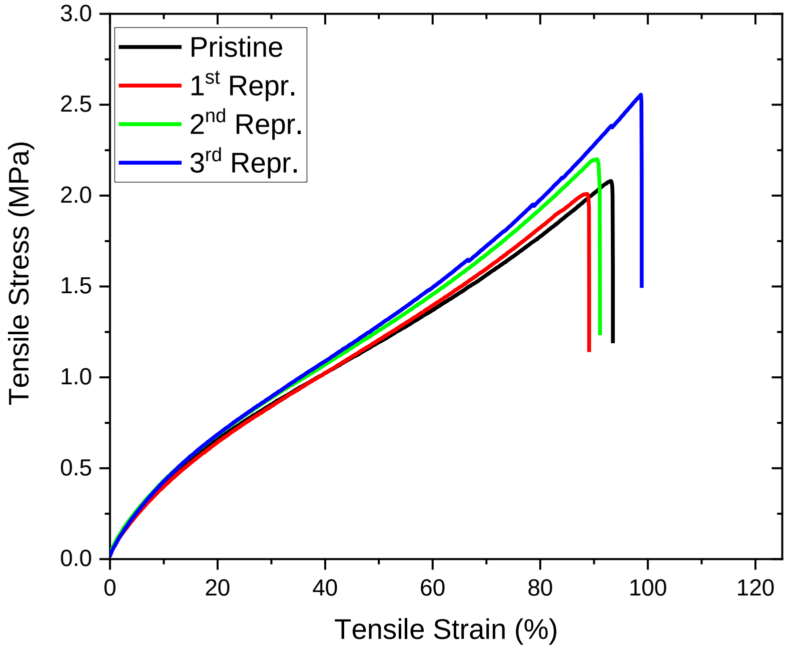

Figure S28. Stress-strain curves of **CAN MeO-Pri$_{1}$-TAEA$_{0}$**.

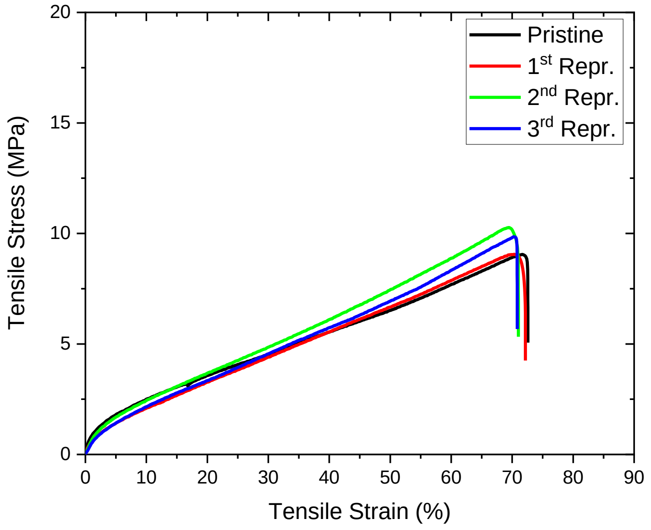

Figure S29. Stress-strain curves of CAN **MeO-Pri$_{0.66}$-TAEA$_{0.33}$.**

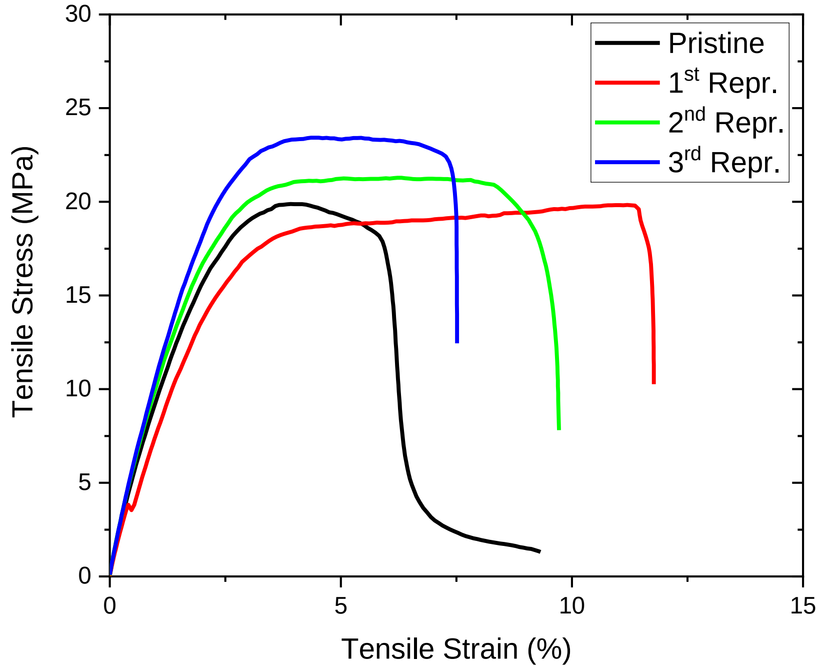

Figure S30. Stress-strain curves of CAN **MeO-Pri$_{0.33}$-TAEA$_{0.66}$.**

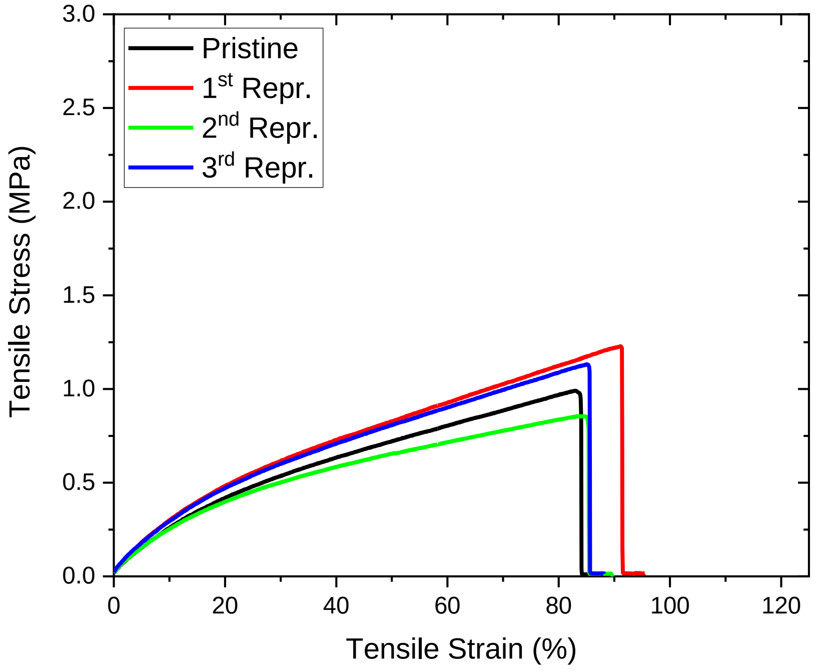

Figure S31. Stress-strain curves of CAN **AllylO-Pri$_1$-TAEA$_0$**.

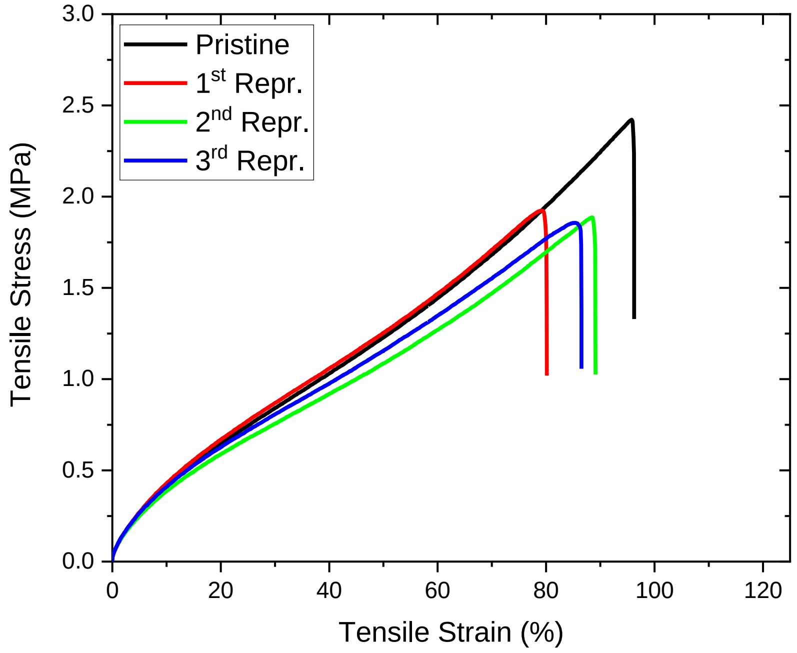

Figure S32. Stress-strain curves of CAN **AllylO-Pri$_{0.66}$-TAEA$_{0.33}$.**

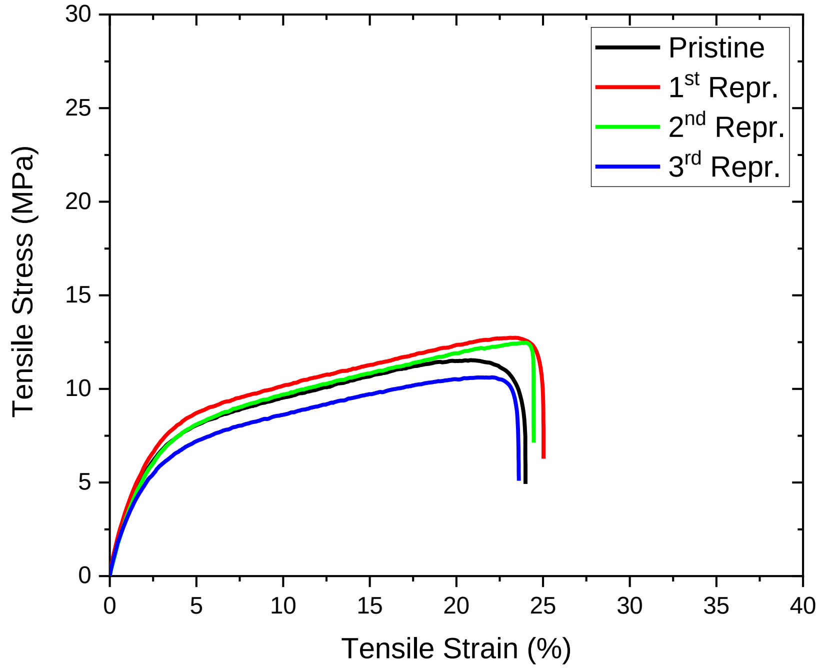

Figure S33. Stress-strain curves of CAN **AllylO-Pri$_{0.33}$-TAEA$_{0.66}$.**

^{1}H NMR spectra of hydrolyzed CAN MeO-Pri$_{0.66}$-TAEA$_{0.33}$

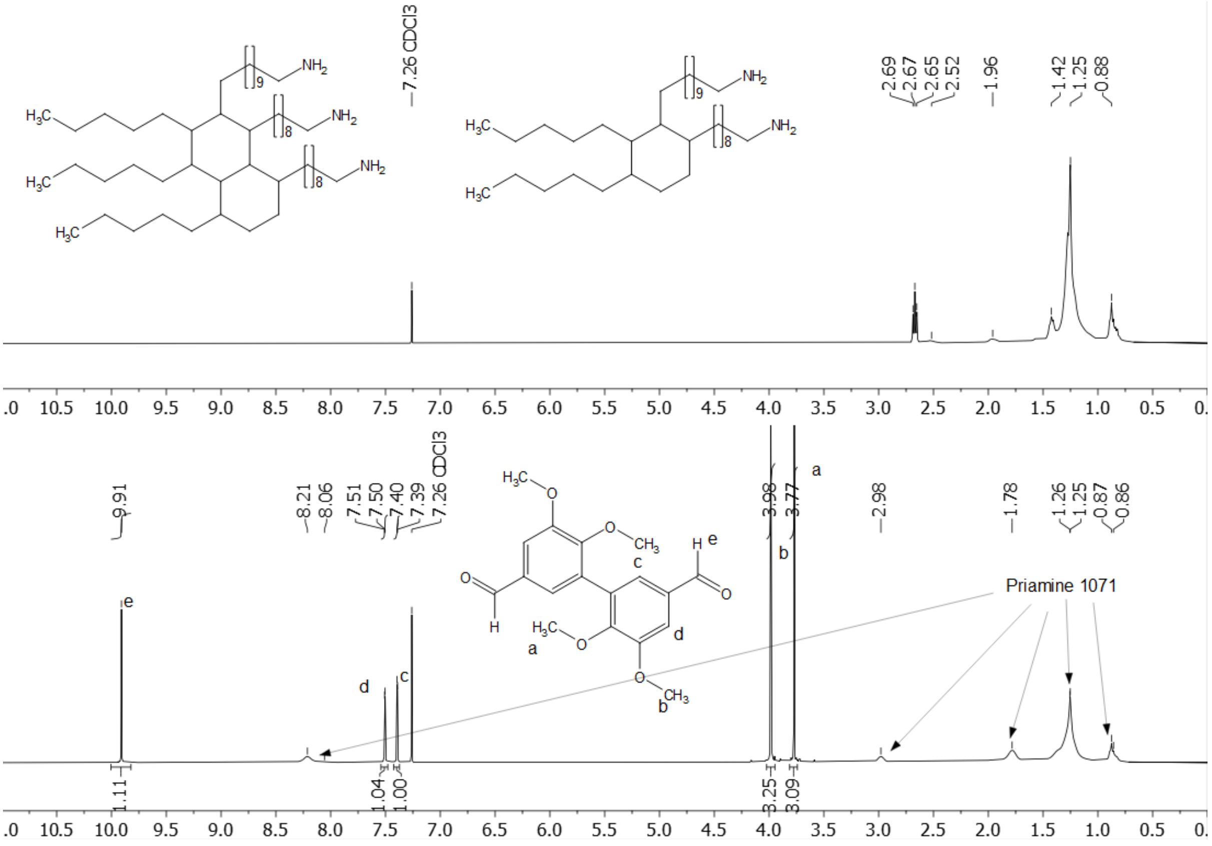

Figure S34. ^{1}H NMR of Praimine 1071 (*top*) ^{1}H NMR of residue after immersing CAN **MeO-Pri$_{0.66}$-TAEA$_{0.33}$** in a solution of an aqueous solution of HCl (1M) at room temperature for 24 h (*bottom*) (400 MHz, 298K, $CDCl_3$).

Calculations for stress relaxation derived from activation energy

- Equation obtained from Arrhenius law
  - **MeO-Pri$_1$-TAEA$_0$** ($Ln\,\tau^* = 6.17\,T^{-1} - 14.68$)
  - **MeO-Pri$_{0.66}$-TAEA$_{0.33}$** ($Ln\,\tau^* = 11.53\,T^{-1} - 30.44$)
  - **MeO-Pri$_{0.33}$-TAEA$_{0.66}$** ($Ln\,\tau^* = 5.91\,T^{-1} - 13.58$)
  - **AllylO-Pri$_1$-TAEA$_0$** ($Ln\,\tau^* = 5.64\,T^{-1} - 13.48$)
  - **AllylO-Pri$_{0.66}$-TAEA$_{0.33}$** ($Ln\,\tau^* = 8.73\,T^{-1} - 23.62$)
  - **AllylO-Pri$_{0.33}$-TAEA$_{0.66}$** ($Ln\,\tau^* = 4.76\,T^{-1} - 11.25$)
- Arrhenius law related to E_a

$$Ln\,\tau^* = \tau_0\,e^{\frac{E_a}{RT}} \quad (R = 8.314\,\frac{J}{mol\,K})$$

$$Ln\,\tau^* = Ln\,\tau_0 + \frac{E_a}{RT} \quad (R = 8.314\,\frac{J}{mol\,K})$$

  - **MeO-Pri$_1$-TAEA$_0$** ($E_a = 6.17 \times 1000 \times 8.314 = 51.3\,kJ/mol$)
  - **MeO-Pri$_{0.66}$-TAEA$_{0.33}$** ($E_a = 11.53 \times 1000 \times 8.314 = 95.9\,kJ/mol$)
  - **MeO-Pri$_{0.33}$-TAEA$_{0.66}$** ($E_a = 5.91 \times 1000 \times 8.314 = 49.1\,kJ/mol$)
  - **AllylO-Pri$_1$-TAEA$_0$** ($E_a = 5.64 \times 1000 \times 8.314 = 46.9\,kJ/mol$)
  - **AllylO-Pri$_{0.66}$-TAEA$_{0.33}$** ($E_a = 8.73 \times 1000 \times 8.314 = 72.6\,kJ/mol$)
  - **AllylO-Pri$_{0.33}$-TAEA$_{0.66}$** ($E_a = 4.76 \times 1000 \times 8.314 = 39.6\,kJ/mol$)

Calculations for vitrimer temperature (T_v) using Arrhenius equation from stress relaxation

- Equation obtained from Arrhenius law
  - **MeO-Pri$_1$-TAEA$_0$** ($Ln\,\tau^* = 6.17\,T^{-1} - 14.68$)
  - **MeO-Pri$_{0.66}$-TAEA$_{0.33}$** ($Ln\,\tau^* = 11.53\,T^{-1} - 30.44$)
  - **MeO-Pri$_{0.33}$-TAEA$_{0.66}$** ($Ln\,\tau^* = 5.91\,T^{-1} - 13.58$)
  - **AllylO-Pri$_1$-TAEA$_0$** ($Ln\,\tau^* = 5.64\,T^{-1} - 13.48$)
  - **AllylO-Pri$_{0.66}$-TAEA$_{0.33}$** ($Ln\,\tau^* = 8.73\,T^{-1} - 23.62$)
  - **AllylO-Pri$_{0.33}$-TAEA$_{0.66}$** ($Ln\,\tau^* = 4.76\,T^{-1} - 11.25$)
- Maxwell equation is used to calculate T_v, using a viscosity value ($\eta = 10^{12}$ Pa·s).

$$\eta = G \times \tau^*$$

$$G^2 = G'^2 + G''^2$$

- G´ and G´´ values were obtained from DMA frequency sweep experiments at room temperature and at 1 Hz.

$$G = \sqrt{G'^2 + G''^2}$$

$$\tau^* = {}^{\eta}/_{G}$$

  - **MeO-Pri$_1$-TAEA$_0$** $(G = 31.34\ MPa)$, $(\tau^* = 31912.4\ MPa)$, $(Ln\ \tau^* = 10.37)$
  - **MeO-Pri$_{0.66}$-TAEA$_{0.33}$** $(G = 89.18\ MPa)$, $(\tau^* = 11213.6\ MPa)$, $(Ln\ \tau^* = 9.32)$
  - **MeO-Pri$_{0.33}$-TAEA$_{0.66}$** $(G = 724.7\ MPa)$, $(\tau^* = 1379.8\ MPa)$, $(Ln\ \tau^* = 7.23)$
  - **AllylO-Pri$_1$-TAEA$_0$** $(G = 3.46\ MPa)$, $(\tau^* = 288803.9\ MPa)$, $(Ln\ \tau^* = 12.57)$
  - **AllylO-Pri$_{0.66}$-TAEA$_{0.33}$** $(G = 7.19\ MPa)$, $(\tau^* = 138993.4\ MPa)$, $(Ln\ \tau^* = 11.84)$
  - **AllylO-Pri$_{0.33}$-TAEA$_{0.66}$** $(G = 451.4\ MPa)$, $(\tau^* = 2215.5\ MPa)$, $(Ln\ \tau^* = 7.70)$

- Stress relaxation equation is used to calculate T_v

MeO-Pri$_1$-TAEA$_0$

$$x = \frac{1000}{T} = \frac{Ln\ \tau^* + 14.68}{6.17} = 4.06$$

$$T_v = \frac{1000}{x} = \frac{1000}{4.06} = 246\ K = -27\ °C$$

MeO-Pri$_{0.66}$-TAEA$_{0.33}$

$$x = \frac{1000}{T} = \frac{Ln\ \tau^* + 30.44}{11.53} = 3.45$$

$$T_v = \frac{1000}{x} = \frac{1000}{3.45} = 290\ K = 17\ °C$$

MeO-Pri$_{0.33}$-TAEA$_{0.66}$

$$x = \frac{1000}{T} = \frac{Ln\ \tau^* + 13.58}{5.91} = 3.52$$

$$T_v = \frac{1000}{x} = \frac{1000}{3.52} = 284\ K = \ 11\ °C$$

AllylO-Pri$_1$-TAEA$_0$

$$x = \frac{1000}{T} = \frac{Ln\ \tau^* + 13.48}{5.64} = 4.62$$

$$T_v = \frac{1000}{x} = \frac{1000}{4.62} = 216\ K = \ -57\ °C$$

AllylO-Pri$_{0.66}$-TAEA$_{0.33}$

$$x = \frac{1000}{T} = \frac{Ln\ \tau^* + 23.62}{8.73} = 4.06$$

$$T_v = \frac{1000}{x} = \frac{1000}{4.06} = 246\ K = \ -27\ °C$$

AllylO-Pri$_{0.33}$-TAEA$_{0.66}$

$$x = \frac{1000}{T} = \frac{Ln\ \tau^* + 11.25}{4.76} = 3.98$$

$$T_v = \frac{1000}{x} = \frac{1000}{3.98} = 251\ K = \ -22\ °C$$

Table S1. Mechanical properties of CANs MeO-Pri$_x$-TAEA$_y$ and AllylO-Pri$_x$-TAEA$_y$.

Entry	CAN	Reprocessing Cycle (RC)	*E* (MPa)	σ_b (MPa)	ε_b (%)
1	**MeO-Pri$_1$-TAEA$_0$**	Pristine	5.76 ± 0.64	2.06 ± 0.28	91.1 ± 6.4
2	**MeO-Pri$_1$-TAEA$_0$**	1st	5.08 ± 0.36	1.90 ± 0.21	87.0 ± 6.0
3	**MeO-Pri$_1$-TAEA$_0$**	2nd	5.46 ± 0.41	2.18 ± 0.36	92.2 ± 10.5
4	**MeO-Pri$_1$-TAEA$_0$**	3rd	5.27 ± 0.14	2.51 ± 0.19	95.2 ± 4.9
5	**MeO-Pri$_{0.66}$-TAEA$_{0.33}$**	Pristine	5.4 ± 8.9	9.2 ± 0.8	73.1 ± 4.9
6	**MeO-Pri$_{0.66}$-TAEA$_{0.33}$**	1st	42.0 ± 8.3	8.9 ± 0.6	70.9 ± 2.7

7	**MeO-Pri$_{0.66}$-TAEA$_{0.33}$**	2nd	62.0 ±4.3	9.7 ± 0.9	70.3 ± 1.9
8	**MeO-Pri$_{0.66}$-TAEA$_{0.33}$**	3rd	52.3 ± 4.5	10.1 ± 0.8	71.5 ± 4.6
9	**MeO-Pri$_{0.33}$-TAEA$_{0.66}$**	Pristine	1065 ± 111	18.7 ± 2.2	5.7 ± 1.6
10	**MeO-Pri$_{0.33}$-TAEA$_{0.66}$**	1st	1039 ± 115	19.1 ± 1.4	10.0 ± 1.4
11	**MeO-Pri$_{0.33}$-TAEA$_{0.66}$**	2nd	1161 ± 114	21.3 ± 2.3	7.8 ± 2.1
12	**MeO-Pri$_{0.33}$-TAEA$_{0.66}$**	3rd	1129 ± 103	22.3 ± 2.4	7.9 ± 2.3
13	**AllylO-Pri$_{1}$-TAEA$_{0}$**	Pristine	3.61 ± 0.34	1.07 ± 0.13	85.5 ± 6.6
14	**AllylO-Pri$_{1}$-TAEA$_{0}$**	1st	3.69 ± 0.09	1.21 ± 0.03	90.8 ± 1.4
15	**AllylO-Pri$_{1}$-TAEA$_{0}$**	2nd	3.65 ± 0.19	1.00 ± 0.11	90.2 ± 4.8
16	**AllylO-Pri$_{1}$-TAEA$_{0}$**	3rd	3.68 ± 0.17	1.15 ± 0.05	87.0 ± 4.0
17	**AllylO-Pri$_{0.66}$-TAEA$_{0.33}$**	Pristine	7.09 ± 0.43	2.61 ± 0.25	94.0 ± 5.8
18	**AllylO-Pri$_{0.66}$-TAEA$_{0.33}$**	1st	6.23 ± 0.52	1.88 ± 0.17	78.9 ± 8.1
19	**AllylO-Pri$_{0.66}$-TAEA$_{0.33}$**	2nd	6.27 ± 0.45	1.93 ± 0.13	87.4 ± 8.4
20	**AllylO-Pri$_{0.66}$-TAEA$_{0.33}$**	3rd	6.31 ± 0.61	1.82 ± 0.28	82.7 ± 10.2
21	**AllylO-Pri$_{0.33}$-TAEA$_{0.66}$**	Pristine	372 ± 32	11.3 ± 1.6	18.8 ± 6.8
22	**AllylO-Pri$_{0.33}$-TAEA$_{0.66}$**	1st	362 ± 19	12.8 ± 0.7	26.3 ± 4.1
23	**AllylO-Pri$_{0.33}$-TAEA$_{0.66}$**	2nd	323 ± 36	11.1 ± 1.1	21.8 ± 5.3
24	**AllylO-Pri$_{0.33}$-TAEA$_{0.66}$**	3rd	344 ± 46	11.1 ± 1.3	23.4 ± 3.9

Table S2. Gel content after immersion in different media for 24 h at r.t of CANs MeO-Pri$_x$-TAEA$_y$ and AllylO-Pri$_x$-TAEA$_y$.

Entry	CAN	Solvent	Gel Content (%)
1	**MeO-Pri$_1$-TAEA$_0$**	THF	64 ± 3
2	**MeO-Pri$_{0.66}$-TAEA$_{0.33}$**	THF	0
3	**MeO-Pri$_{0.33}$-TAEA$_{0.66}$**	THF	0
4	**AllylO-Pri$_1$-TAEA$_0$**	THF	69 ± 4
5	**AllylO-Pri$_{0.66}$-TAEA$_{0.33}$**	THF	0
6	**AllylO-Pri$_{0.33}$-TAEA$_{0.66}$**	THF	0
7	**MeO-Pri$_1$-TAEA$_0$**	EtOH	98 ± 1
8	**MeO-Pri$_{0.66}$-TAEA$_{0.33}$**	EtOH	98 ± 1
9	**MeO-Pri$_{0.33}$-TAEA$_{0.66}$**	EtOH	99 ± 1
10	**AllylO-Pri$_1$-TAEA$_0$**	EtOH	98 ± 1
11	**AllylO-Pri$_{0.66}$-TAEA$_{0.33}$**	EtOH	96 ± 2
12	**AllylO-Pri$_{0.33}$-TAEA$_{0.66}$**	EtOH	98 ± 1
13	**MeO-Pri$_1$-TAEA$_0$**	DMF	97 ± 1
14	**MeO-Pri$_{0.66}$-TAEA$_{0.33}$**	DMF	97 ± 2
15	**MeO-Pri$_{0.33}$-TAEA$_{0.66}$**	DMF	99 ± 1
16	**AllylO-Pri$_1$-TAEA$_0$**	DMF	96 ± 1
17	**AllylO-Pri$_{0.66}$-TAEA$_{0.33}$**	DMF	93 ± 4
18	**AllylO-Pri$_{0.33}$-TAEA$_{0.66}$**	DMF	95 ± 1

19	**MeO-Pri$_1$-TAEA$_0$**	Distilled water	99 ± 1
20	**MeO-Pri$_{0.66}$-TAEA$_{0.33}$**	Distilled water	99 ± 1
21	**MeO-Pri$_{0.33}$-TAEA$_{0.66}$**	Distilled water	99 ± 1
22	**AllylO-Pri$_1$-TAEA$_0$**	Distilled water	99 ± 1
23	**AllylO-Pri$_{0.66}$-TAEA$_{0.33}$**	Distilled water	99 ± 1
24	**AllylO-Pri$_{0.33}$-TAEA$_{0.66}$**	Distilled water	99 ± 1
25	**MeO-Pri$_1$-TAEA$_0$**	$NaOH_{(aq)}$ (1M)	99 ± 1
26	**MeO-Pri$_{0.66}$-TAEA$_{0.33}$**	$NaOH_{(aq)}$ (1M)	99 ± 1
27	**MeO-Pri$_{0.33}$-TAEA$_{0.66}$**	$NaOH_{(aq)}$ (1M)	99 ± 1
28	**AllylO-Pri$_1$-TAEA$_0$**	$NaOH_{(aq)}$ (1M)	99 ± 1
29	**AllylO-Pri$_{0.66}$-TAEA$_{0.33}$**	$NaOH_{(aq)}$ (1M)	99 ± 1
30	**AllylO-Pri$_{0.33}$-TAEA$_{0.66}$**	$NaOH_{(aq)}$ (1M)	99 ± 1
31	**MeO-Pri$_1$-TAEA$_0$**	$HCl_{(aq)}$ (1 M)	n.a
32	**MeO-Pri$_{0.66}$-TAEA$_{0.33}$**	$HCl_{(aq)}$ (1 M)	n.a
33	**MeO-Pri$_{0.33}$-TAEA$_{0.66}$**	$HCl_{(aq)}$ (1 M)	n.a
34	**AllylO-Pri$_1$-TAEA$_0$**	$HCl_{(aq)}$ (1 M)	n.a
35	**AllylO-Pri$_{0.66}$-TAEA$_{0.33}$**	$HCl_{(aq)}$ (1 M)	n.a
36	**AllylO-Pri$_{0.33}$-TAEA$_{0.66}$**	$HCl_{(aq)}$ (1 M)	n.a

Characterization of recovered CAN AllylO-Pri$_{0.33}$-TAEA$_{0.66}$

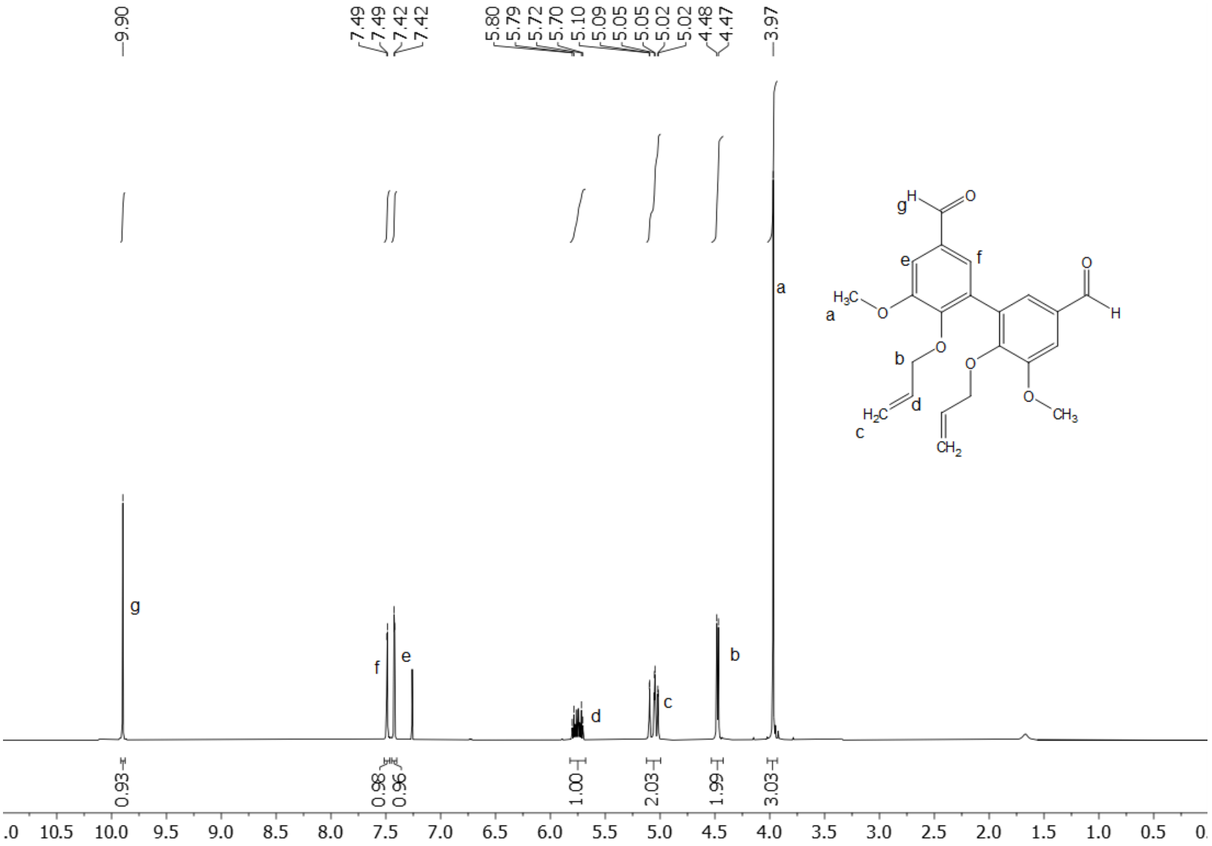

Figure S35. ^{1}H NMR of isolated **Di-Van-OAllyl** after acidic hydrolysis of CAN **AllylO-Pri$_{0.33}$-TAEA$_{0.66}$** (400 MHz, 298K, $CDCl_3$).

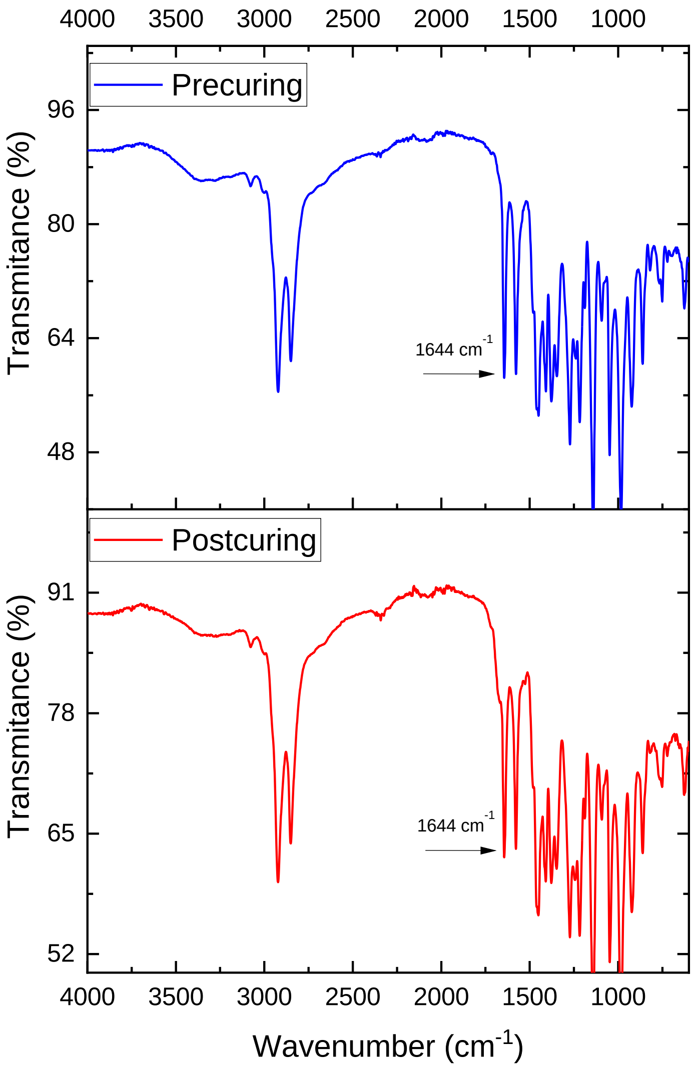

Figure S36. FTIR spectrum of recycled network **AllylO-Pri$_{0.33}$-TAEA$_{0.66}$** before curing process (*top*), FTIR spectrum of recycled network **AllylO-Pri$_{0.33}$-TAEA$_{0.66}$** after curing process 140 °C during 8 h (*bottom*).
